# Biotransformation of (–)-Isopulegol by *Rhodococcus rhodochrous*

**DOI:** 10.3390/ph15080964

**Published:** 2022-08-03

**Authors:** Irina B. Ivshina, Natalia A. Luchnikova, Polina Yu. Maltseva, Irina V. Ilyina, Konstantin P. Volcho, Yurii V. Gatilov, Dina V. Korchagina, Nadezhda A. Kostrikina, Vladimir V. Sorokin, Andrey L. Mulyukin, Nariman F. Salakhutdinov

**Affiliations:** 1 Institute of Ecology and Genetics of Microorganisms of the Ural Branch of the Russian Academy of Sciences, Perm Federal Research Center of the Ural Branch of the Russian Academy of Sciences, 13 Golev Str., 614081 Perm, Russia; luchnikova.n@mail.ru; 2 Department of Microbiology and Immunology, Perm State National Research University, 15 Bukirev Str., 614990 Perm, Russia; inbox.98@bk.ru; 3 N.N. Vorozhtsov Novosibirsk Institute of Organic Chemistry of the Siberian Branch of the Russian Academy of Sciences, 9 Lavrentiev Avenue, 630090 Novosibirsk, Russia; ilyina@nioch.nsc.ru (I.V.I.); volcho@nioch.nsc.ru (K.P.V.); gatilov@nioch.nsc.ru (Y.V.G.); verbenon@mail.ru (D.V.K.); anvar@nioch.nsc.ru (N.F.S.); 4 Winogradsky Institute of Microbiology, Research Center of Biotechnology, Russian Academy of Sciences, 60 let Oktyabrya, 7, bld. 2, 117312 Moscow, Russia; nadin-kost@yandex.ru (N.A.K.); vlvlsorokin@gmail.com (V.V.S.); andlm@mail.ru (A.L.M.)

**Keywords:** biotransformation, halloysite nanotubes, (–)-isopulegol, monoterpenoid, *Rhodococcus rhodochrous*

## Abstract

The ability of actinobacteria of the genus *Rhodococcus* to biotransform the monoterpenoid (–)-isopulegol has been established for the first time. *R*. *rhodochrous* strain IEGM 1362 was selected as a bacterium capable of metabolizing (–)-isopulegol to form new, previously unknown, 10-hydroxy (**2**) and 10-carboxy (**3**) derivatives, which may presumably have antitumor activity and act as respiratory stimulants and cancer prevention agents. In the experiments, optimal conditions were selected to provide the maximum target catalytic activity of rhodococci. Using up-to-date (TEM, AFM-CLSM, and EDX) and traditional (cell size, roughness, and zeta potential measurements) biophysical and microbiological methods, it was shown that (–)-isopulegol and halloysite nanotubes did not negatively affect the bacterial cells. The data obtained expand our knowledge of the biocatalytic potential of rhodococci and their possible involvement in the synthesis of pharmacologically active compounds from plant derivatives.

## 1. Introduction

Given the need for highly effective pharmacological agents for the treatment of socially significant diseases, the pressing task is to synthesize novel chemical compounds with potential biological activities, including those derived from plant terpenoids [1]. These compounds are of particular interest for the synthesis of substances with antiviral, antimicrobial, anti-inflammatory, antitumor, and neuroprotective activities [2,3].

**Figure 1:**
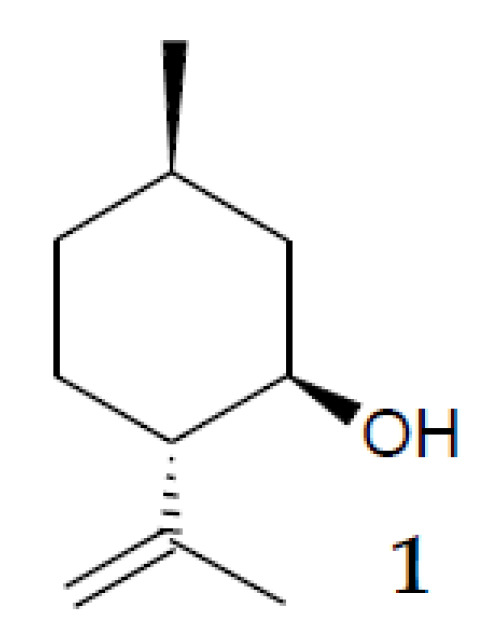
Structure 1: (–)-isopulegol.

In recent years, increased attention of synthetic chemists has been drawn to the monoterpene alcohol (–)-isopulegol (**1**, (1*R*,3*R*,4*S*)-*p*-menth-8-en-3-ol, C_10_H_18_O, Figure 1), due to its low cost, availability and various properties [4,5]. Chemical methods of transformation of (–)-isopulegol are helpful for obtaining compounds with pronounced antiviral [6], analgesic [7], antioxidant, antimicrobial [8], and antiproliferative activities [9]. However, chemical synthesis of bioactive derivatives of (–)-isopulegol requires expensive (often aggressive) reagents as well as the introduction of protective groups of reactive functional centers of the molecule [10]. At the same time, the conversion of (–)-isopulegol may frequently result in a difficult-to-separate mixture of products. The biological catalysis creates opportunities for obtaining target products based on the principles of green and sustainable pharmacy, i.e., at normal temperatures, pressures, and pH; in environmentally friendly conditions; and with a minimal yield of by-products [11,12,13,14].

So far, only single studies have been carried out on the biocatalytic transformation of (–)-isopulegol. Possible biotransformations of this compound by immobilized cutinases from *Aspergillus oryzae* and *Humicola insolens* and lipases from *Thermomyces lanuginosus* and *Rhizomucor miehei* have been investigated [15]. However, application of immobilized enzyme systems compared to whole microbial cells is an extremely labor- and time-consuming process that involves expensive specialized equipment [16].

Members of the genus *Rhodococcus* are biotechnologically significant microorganisms. They are promising for biocatalytic transformations of various hydrophobic organic compounds due to biosurfactants produced and polytrophic and labile metabolic systems [17,18]. According to our data, different strains of rhodococci from the Regional Specialised Collection of Alkanotrophic Microorganisms (acronym IEGM, www.iegmcol.ru, accessed on 6 April 2022), utilize plant tricyclic diterpenoids and pentacyclic triterpenoids with the formation of bioactive derivatives [19,20]. As for (–)-isopulegol, *Rhodococcus* spp. was first shown to use this compound as a sole carbon source in a single paper [21]. However, it was not the objective of that study to analyze the bioconversion products of this monoterpenoid. The IEGM collection of numerous and diverse strains of rhodococci may very likely harbor effective (–)-isopulegol-biotransforming strains.

Biotransformation of poorly metabolized organic compounds by microbial cells is often a long process; therefore, it seems interesting to use nanomaterials [22], for example, halloysite nanotubes (HNTs, Al_2_Si_2_O_5_(OH)_4_). Natural clay mineral halloysite consists of tetrahedral SilAO and octahedral AlAO sheets. HNTs have found use as nano-cargos for drug delivery, adsorbents and components of composite materials [23,24]. However, most importantly, HNTs contribute to the improved biodegradation activity of filamentous fungus *Aspergillus fumigates* [25] and bacterium *Bacillus licheniformis* [26], and enhance bacterial cellulose production by *Gluconacetobacter xylinus* [27]. However, HNTs exhibit antibacterial and bacteriostatic activity against a number of bacteria [28,29], a fact that should be considered when developing biocatalytic technologies involving particular microorganisms.

The objective of this study was to investigate the process of biotransformation of (–)-isopulegol using *Rhodococcus* spp. as well as to search for an active strain, to select optimal growth and development conditions, and to analyze the resulting products and their bioactivity.

## 2. Results and Discussion

### 2.1. Screening for a Target Rhodococcus Strain and Evaluation of Its Response to (–)-Isopulegol and/or HNTs

The primary screening included 40 IEGM strains, and only four of them, viz., *Rhodococcus opacus* (1 strain) and *R*. *rhodochrous* (3 strains), were able to utilize (–)-isopulegol as the only carbon and energy source. The strain selected for further research was *R*. *rhodochrous* IEGM 1362 isolated from peat deposits (Paltinskoye peat deposit, Perm Krai, Russia). The strain proved to be highly effective in degrading (–)-isopulegol.

*R*. *rhodochrous* IEGM 1362 showed good growth on nutrient-rich agar media and liquid nutrient BTN-based media. Mineral RS medium with (–)-isopulegol as the only carbon source supported only slow growth of this strain and low yield of biomass due to the lack of growth factors and substrates. Better growth was provided by adding yeast extract to this medium. “Compatibility” tests for growing cultures of *R*. *rhodochrous* IEGM 1362 with (–)-isopulegol and HNTs in different nutrient media showed the results discussed below.

**Figure 2:**
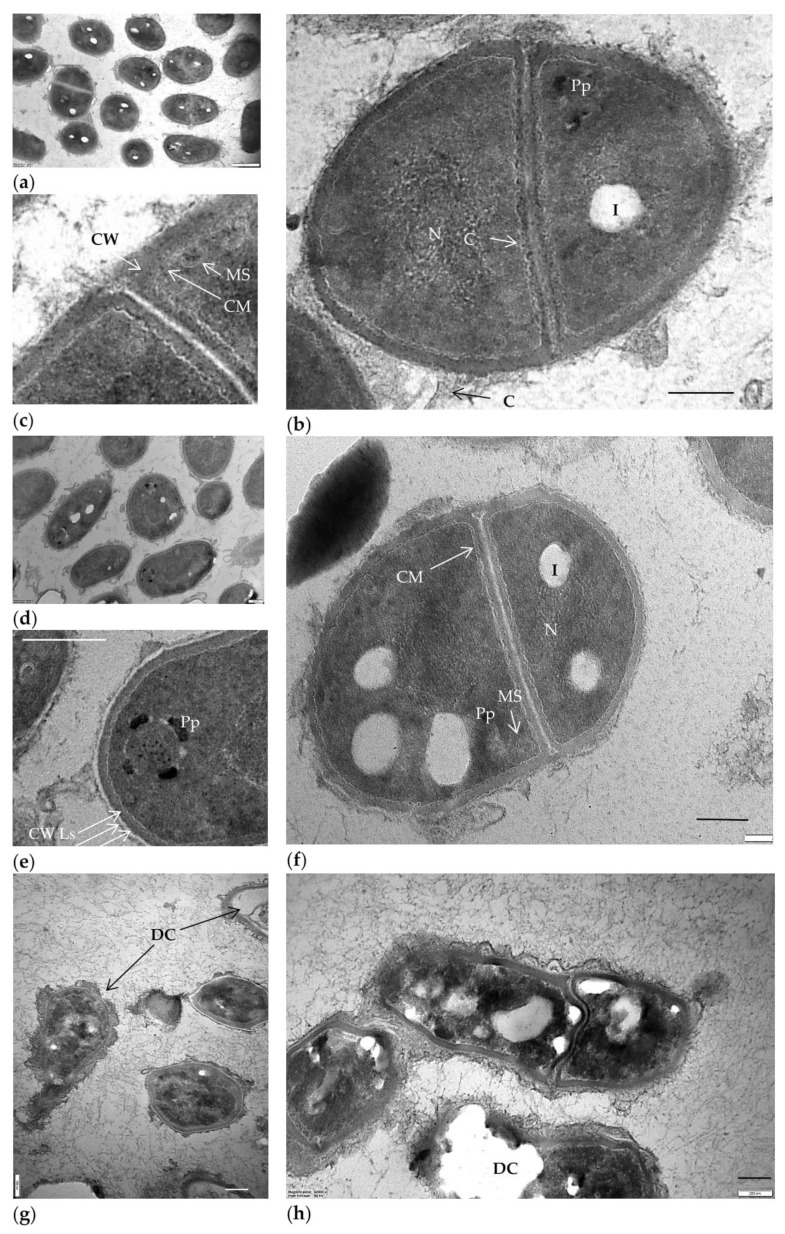
Thin sections of *R. rhodochrous* IEGM 1362 as viewed with TEM in cultures grown on nutrient agar (**a**–**c**) without (control) or (**d**–**f**) with (–)-isopulegol (0.025% *v*/*v*) and (**g**,**h**) in acute tests with the addition of (–)-isopulegol onto agar with already grown culture. Designations: C, capsular layer; CW, cell wall; CW Ls, cell wall layers; CM, cytoplasmic membrane; N, nucleoid; Pp, polyphosphate particles; L, lipid inclusions; MS, membrane-like structures; DC, destructed cells. Bar, 200 nm.

**Figure 3:**
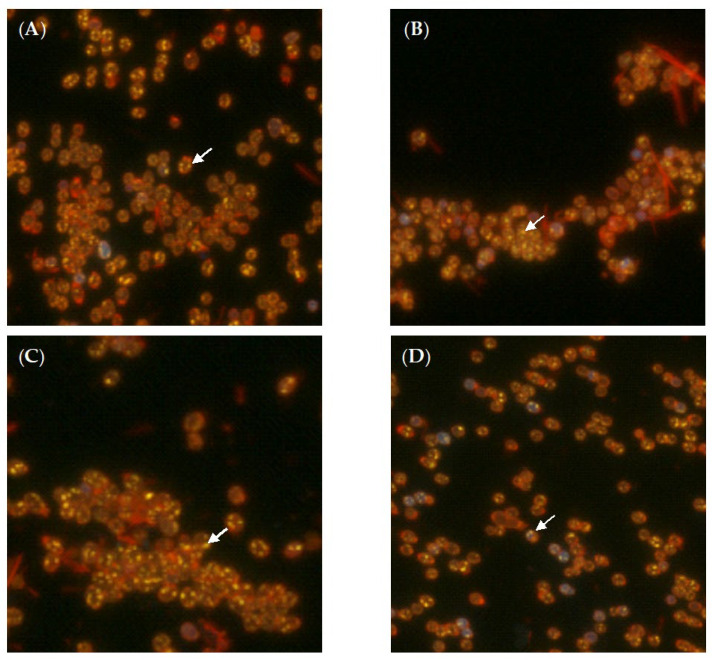
Fluorescent microscopy (1000×) of *R*. *rhodochrous* IEGM 1362 cells stained with Nile Red: (**A**)—biotic control, (**B**)—in the presence of (–)-isopulegol, (**C**)—HNTs, (**D**)—(–)-isopulegol and HNTs. Arrows indicate lipid inclusions.

No cytotoxic effect on cells was detected in cultures of the test strain grown on BTN agar in the presence of (–)-isopulegol (0.025% *v*/*v*) [21]. Thus, comparative TEM-based analysis of ultrathin sections showed no sharp differences in the morphology of cells grown with (Figure 2d–f) and without 0.025% (*v*/*v*) of (–)-isopulegol (Figure 2a–c). Cells in both variants were intact and had no signs of lysis or deformation (Figure 2a,d). The dividing cells possessed the outer capsular layer (visible as outgrowths), the cell wall with layers of varying electron density (common for rhodococci), and the intact cytoplasmic membrane. The nucleoid, a few electron-dense particles (probably composed of polyphosphates), and electron-transparent lipid inclusions were easily discernible in the finely textured cytoplasm, as well as spherical membrane-like structures (Figure 2e). Intracellular lipids were also detectable with epifluorescence microscopy after staining with Nile Red (Figure 3). Some cells in cultures grown with (–)-isopulegol had a more stratified cell wall and polyphosphate particles on spheres (see Figure 2e), thus slightly differing from the control cultures. In addition, we showed that the cell morphology of *R. rhodochrous* IEGM 1362 grown on the medium with HNTs (0.1 g/L) or (–)-isopulegol (0.025% *v*/*v*) and HNTs (0.1 g/L) was similar to that in the control (not shown here). The addition of (–)-isopulegol onto agar with already grown cultures in acute tests (to mimic high concentrations) caused severe destructive and cytotoxic effects on cells (see Figure 2g,h).

**Figure 4:**
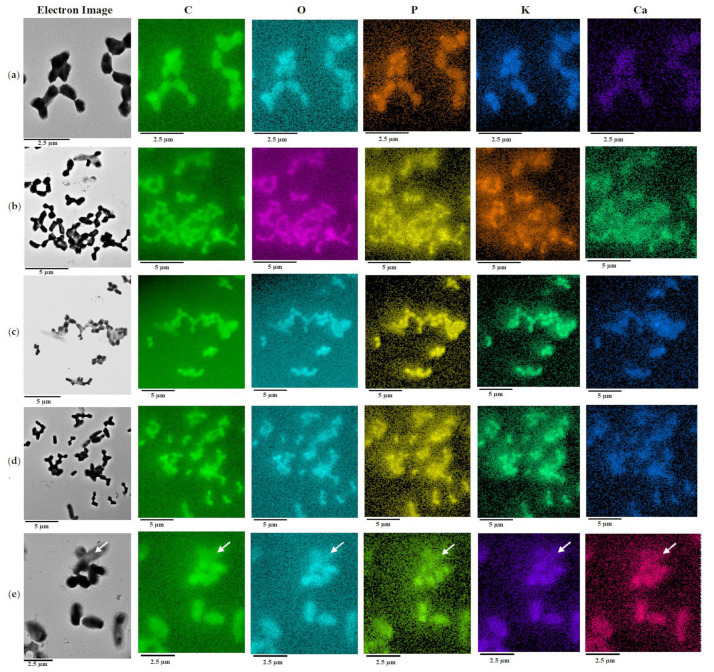
Elemental composition of *R*. *rhodochrous* IEGM 1362 determined by mapping using X-ray microanalysis. Distribution of individual chemical elements is highlighted with color. Cell growth on: (**a**)—BTN agar, (**b**)—BTN agar plus (–)-isopulegol, (**c**)—BTN agar plus HNT, (**d**)—BTN agar plus (–)-isopulegol and HNTs, or (**e**)—in RS medium with (–)-isopulegol as the only carbon and energy source. The white arrow indicates a cell depleted in the set of elements and, apparently, inactive or dead.

TEM-EDX spectral analysis and elemental mapping of unfixed cells showed that additions of (–)-isopulegol (0.025% *v*/*v*) and/or HNTs (0.1 g/L) to BTN medium caused no changes in the intracellular pool and distribution of C, O, P, and K (Figure 4, rows a–d). Some (–)-isopulegol-grown cells were more enriched in Ca than those in the control cultures (without additives). The well-detectable K content (Figure 4) points to the integrity of cytoplasmic membranes and the active physiological state, as has been already demonstrated upon comparative TEM-EDX analyses of elements in growing, dormant, and inactive cells [30]. Additionally, complete leakage of K due to disruption of cytoplasmic membranes in rhodococci after treatment with a fixative agent was observed in this study (not shown here). It is noteworthy that there was a heterogeneous population of *R. rhodochrous* IEGM 1362 grown on minimal RS medium with (–)-isopulegol (0.025% *v*/*v*) as the only carbon and energy source. Some cells had a depleted pool of these elements (Figure 4, row e, white arrow), whereas the others were normal. A reduced or too poor intracellular pool of biologically important elements may suggest an inactive or moribund state, as proposed in the above cited work.

Thus, the results of this part of the study showed the absence of any toxic effect of (–)-isopulegol (0.025% *v*/*v*) and HNTs (0.1 g/L) present in the growth medium, individually or in combination.

The development or scaling of the process of biodegradation of (–)-isopulegol and other compounds requires taking into account their possible cytotoxic effects when exceeding the concentration threshold or using another method for their introduction, as was the case in the acute test with the strain under study. The physiological activity of *R*. *rhodochrous* IEGM 1362 on minimal RS medium is lower than on nutrient media, as evident from the results of comparative elemental analysis. However, large amounts of organic substrate (e.g., in nutrient media) can hamper analyzing the bioconversion products of the target compound. Therefore, following the principle of the golden mean, RS medium with small amounts of yeast extract (0.1 g/L) and non-toxic concentrations of (–)-isopulegol and/or HNTs for *R*. *rhodochrous* IEGM 1362 were chosen for further research.

### 2.2. Biotransformation of (–)-Isopulegol by R. rhodochrous IEGM 1362: The State and Activity of Cells

(–)-Isopulegol (0.025% *v*/*v*) and/or HNTs (0.1 g/L) added to the medium did not significantly affect the size of *R*. *rhodochrous* IEGM 1362 cells (Table S1), the cell surface roughness, or electrokinetic potential (Table S2). *R*. *rhodochrous* is known for its resistance to metal nanoparticles [31]. A slight change in roughness and zeta potential of the cell surface in the presence of nickel nanoparticles has been recorded for another strain—IEGM 1363. Moreover, the number of intact cells of *R. rhodochrous* IEGM 1362 (green fluorescence, live/dead test) did not significantly differ in the control medium (98.6% of the total number of cells) and in the medium with (–)-isopulegol and HNTs (93.6 to 97.2% of the total number of cells). This result correlated with the electron microscopy data.

**Figure 5:**
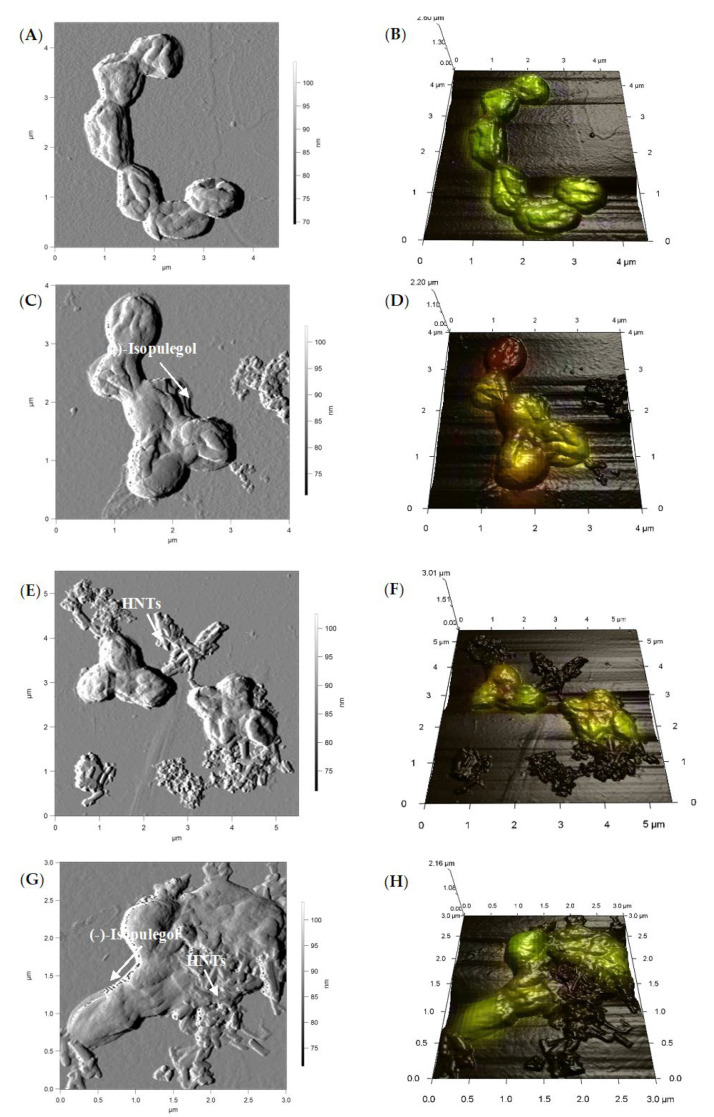
AFM and combined AFM-CLSM images of *R*. *rhodochrous* IEGM 1362 (biotic control **A**, **B**) in the presence of (–)-isopulegol (**C**,**D**), HNTs (**E**,**F**), and (–)-isopulegol and HNTs (**G**,**H**). Green fluorescence indicates living cells, red fluorescence indicates dead cells.

AFM images (Figure 5A–D) and combined AFM-CLSM images (Figure 5A1–D1) revealed the accumulation of droplets of (–)-isopulegol on the surface of bacterial cells (Figure 5B,D) similar to that observed in the biotransformation of terpenoid betulin using rhodococci [19]. Apparently, the process of oxidative bioconversion of terpene substrates involves enzymes of the CYP450-dependent oxygenase group. These enzymes are generally associated with a membrane and require direct contact of cells with the substrate [32]. Thus, the contact area between the *Rhodococcus* cell wall and substrate is a site of accumulation of terpenoid that provides the substrate supply to oxidative enzyme systems. Accumulation of HNTs directly adjacent to *Rhodococcus* cells was also found (Figure 5C,D). In earlier studies, a close contact between HNTs and fungal pellets presumably due to electrostatic and dipole–dipole interactions was shown [25].

**Figure 6:**
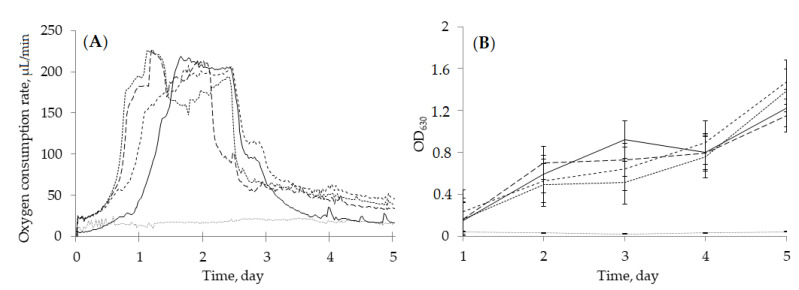
Oxygen consumption rate (**A**) and growth dynamics (**B**) of R. rhodochrous IEGM 1362 in the presence of (‒)-isopulegol and HNTs (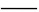), (‒)-isopulegol (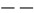), and HNTs (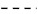). Biotic control (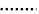), abiotic control (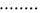).

The growth dynamics of cultures and the metabolic activity of cells were similar in the control and in the presence of (–)-isopulegol and HNTs, as evidenced by formazane formation and OD_630_ measurements (Figure 6B). The maximum respiratory activity of rhodococcal cells was observed for the first 3 days, irrespective of the composition of the culture medium (yeast extract-supplemented RS medium with/without (–)-isopulegol and/or HNTs) (Figure 6A), and coincided with the maximum catalytic activity of bacteria towards (–)-isopulegol. Addition of HNTs, both with (–)-isopulegol and individually, slightly (for less than 12 h) inhibited the respiratory activity of cells, which may be explained by a short adaptation period of bacterial cells to nanomaterial [33]. In the abiotic control (without inoculation of the medium), the respiration values, which determine the biocatalytic nature of substrate oxidation, were almost zero.

**Figure 7:**
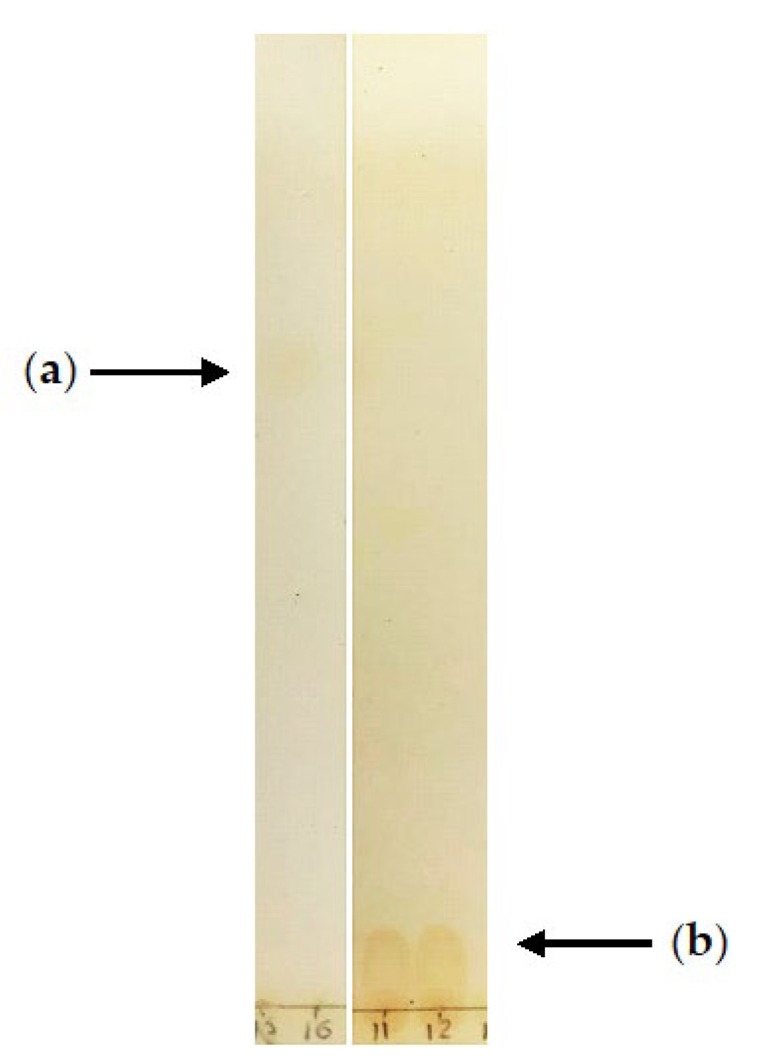
TLC of (–)-isopulegol (**a**) Rf 0.65 and its biotransformation products; (**b**) Rf 0.01 and 0.07 using *R*. *rhodochrous* IEGM 1362 cells.

**Figure 8:**
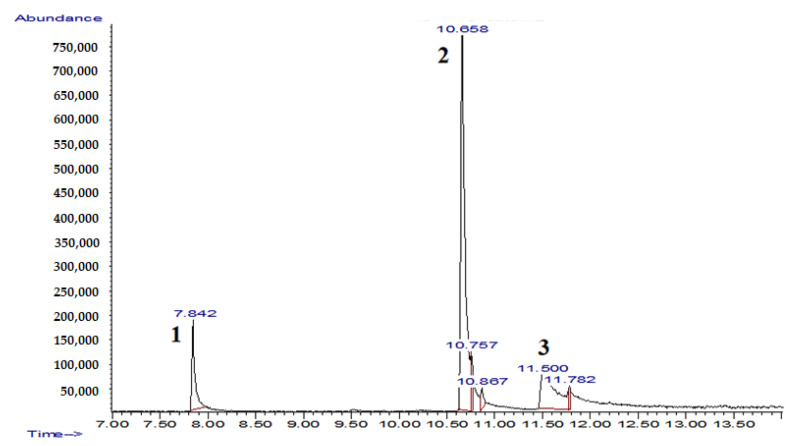
Chromatogram of the extract obtained during biotransformation of (–)-isopulegol (5 days) using *R. rhodochrous* IEGM 1362 in the presence of HNTs: **1**, (–)-isopulegol, **2**, metabolite *m/z* 170, **3**, metabolite *m/z* 184.

The experimental conditions, i.e., the selected strain, medium composition, and supplements proved to be optimal for (–)-isopulegol transformation to form the compounds. After extraction from the post-culture fluid and TLC analysis, the compounds were separated into fractions with Rf 0.01 and 0.07. No residual (–)-isopulegol was detected (Figure 7). GC-MS analysis of extracts obtained on the fifth day of the experimental biotransformation of (–)-isopulegol in the presence of HNTs revealed two previously unidentified derivatives of (–)-isopulegol with *m/z* 170 (**2**) and *m/z* 184 (**3**) (Figure 8).

An extensive GC-MS analysis of daily sampled extracts obtained during the biotransformation of (–)-isopulegol showed the bioconversion process to be dependent on the presence of HNTs and the volume of the medium (Table 1). Thus, in a small volume of HNT-free medium (25 mL), the conversion of (–)-isopulegol **1** by *R. rhodochrous* IEGM 1362 led to the formation of compound **2** after 2 days. The latter was completely converted to compound **3** on the third day of cultivation. The following (4th and 5th) days were marked by increased content of product **3** and enhanced conversion of (–)-isopulegol **1**. Apparently, the initially formed compound **2** underwent further oxidation into compound **3**. At the same time, the addition of HNTs in the transformation medium (25 mL) slightly slowed down the bioconversion process. The highest content of derivative **2** was observed after 3 days of the experiment. On the following days, the selectivity for compound **2** decreased with a simultaneous increase in the selectivity for product **3**.

It is worth noting that the process of monoterpenoid biotransformation in a larger volume of the medium (100 mL) did not depend on the presence of HNTs and was accompanied by a complete conversion of (–)-isopulegol **1** to compound **3** already on the second day of cultivation. Such enhancement of the biotransformation process with an increase in the volume of the cultivation medium may be associated with the features of mass transfer and gas–liquid oxygen transfer capacity per liquid volume and time [34].

### 2.3. Identification of Products of (–)-Isopulegol Biotransformation

**Figure 9:**
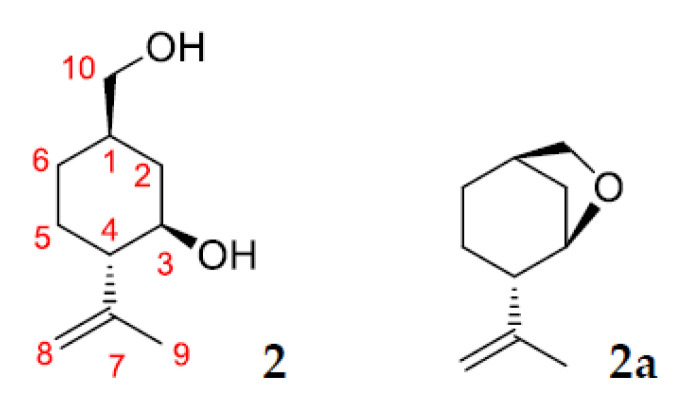
Structures **2** and **2a**.

Compound **2** was isolated by column chromatography on SiO_2_ as white powder (Rf value of 0.01 (hexane/ethyl acetate 1:1)). This compound is a hydroxyl containing (–)-isopulegol derivative, which was confirmed by the presence of the molecular ion peak at *m/z* 170 in the GC-MS and NMR spectral data. Structure **2a** as an alternative of compound **2** was rejected based on the ^13^C-NMR spectrum of CDCl_3_ solution with a D_2_O addition and HMBC spectral data. The presence of hydroxyl groups at C-3 and C-10 was confirmed by D_2_O addition experiment. Due to the isotope effect in this solution, replacement of OH by OD led to upfield shifts of the doublet at 70.06 ppm and triplet at 67.77 ppm (∆δ 0.10 and 0.19 ppm, respectively). The absence of cross-peaks H-3/C-10 and H-10/C-3 in HMBC spectrum also confirms structure **2** for this compound (Figure 9).

**Figure 10:**
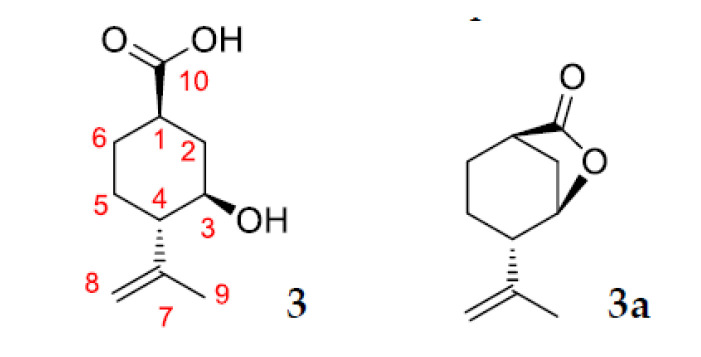
Structures **3** and **3a**.

Compound **3** was isolated by column chromatography on SiO_2_ as white powder (Rf value of 0.07 (hexane/ethyl acetate 1:1)). This compound is a product of further oxidation of diol **2** and contains an acid group at C-10. Structure elucidation of compound **3** was made on the basis with NMR and HR-MS spectral data and confirmed by the presence of the molecular ion peak at *m/z* 184 in GC-MS. Structure **3a** as an alternative of compound **3** was rejected based on the ^13^C-NMR spectrum of CDCl_3_ solution with a D_2_O addition and HMBC spectral data. The presence of a hydroxyl group at C-3 and a carboxyl group at C-10 was confirmed by D_2_O addition experiment. Due to the isotope effect in this solution, replacement of OH by OD led to upfield shifts of the doublet at 70.06 ppm and triplet at 67.77 ppm (∆δ 0.68 and 0.08 ppm, respectively). The absence of cross-peaks H-3/C-10 and H-10/C-3 in HMBC spectrum also confirms structure **3** for this compound (Figure 10).

The structures of compounds **2** and **3** were unambiguously confirmed by X-ray crystallography, which was recorded on Bruker Kappa APEX II equipment. Crystallographic data of compounds **2** and **3** were deposited at the Cambridge Crystallographic Data Centre (CCDC) with deposition numbers of CCDC 2168968 and 2168969, respectively. These data can be obtained free of charge from the CCDC via http://www.ccdc.cam.ac.uk/conts/retrieving.html, accessed on 6 April 2022 (or from the CCDC, 12 Union Road, Cambridge CB2 1EZ, UK; Fax: +44-1223-336033; E-mail: deposit@ccdc.cam.ac.uk). To the best of our knowledge, compounds **2** and **3** have not been previously described in the literature.

**Figure 11:**
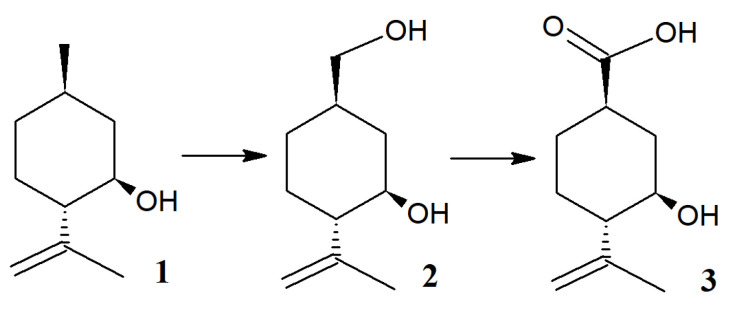
Proposed pathway of (–)-isopulegol bioconversion by *R*. *rhodochrous* IEGM 1362.

Based on GC-MS and NMR spectra of the obtained compounds, we have proposed a scheme for (–)-isopulegol conversion using *R*. *rhodochrous* IEGM 1362 (Figure 11).

**Figure 12:**
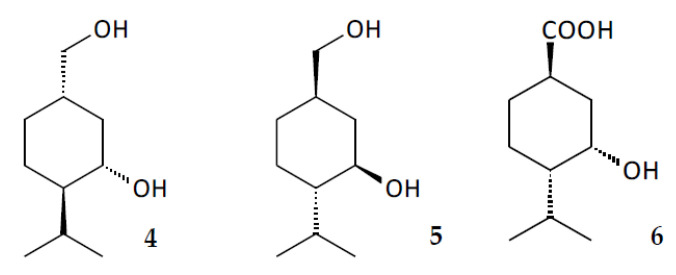
Structures **4**, **5** and **6**.

Similar transformations have been previously documented in the conversion of analogous monoterpenoids using mycelial fungi and Gram-negative bacteria (Figure 12). Thus, 10-hydroxy derivatives of (+)- and (–)-menthol (compounds **4** and **5**, respectively) were obtained using the fungal species *Phomopsis amygdale* [35] and *Aspergillus* [36], while oxidation of (–)-menthone by *Pseudomonas fluorescens* resulted in the formation of its 10-carboxy derivative (compound **6**) [37].

### 2.4. In Silico Analysis of (–)-Isopulegol and Its Derivatives

The performed in silico analysis could suggest the formation of less ecotoxic and hydrophobic compounds than the initial (–)-isopulegol subjected to bioconversion (Table 2). Thus, compared to (–)-isopulegol, the estimated acute toxicity of compounds **2** and **3** was 6.9–9.8 and 85.8–154.1 times lower, respectively, while their chronic toxicity was 4.8–8.7 and 66.6–134.5 times lower, respectively. Moreover, the solubility of compounds **2** and **3** in water may be 1.1 and 20.4 times higher, respectively.

The assessment of biological potential demonstrated that compounds **2** and **3**, unlike (–)-isopulegol, can be used with high probability coefficients as respiratory analeptics (probability coefficients (P_a_) 0.568 and 0.686, respectively), agents for the prevention of bladder cancer (Pa 0.670 and 0.714, respectively) and have antitumor activity (P_a_ 0.550 and 0.482, respectively) (Table 3). Similarly to (–)-isopulegol, the obtained compounds may have carminative (P_a_ 0.928–0.976), anti–eczema (P_a_ 0.908–0.929), and anti–inflammatory (P_a_ 0.731–0.748) activities, act as immunosuppressants (P_a_ 0.720–0.755) and muscle relaxants (P_a_ 0.677–0.751), and also induce the expression of immune response genes (P_a_ 0.730–0.747). The data obtained in silico determine the prospects for further in-depth study of the obtained metabolites, including their biological activity in vitro and in vivo.

## 3. Materials and Methods

### 3.1. Microorganisms

Initial screenings included 40 *Rhodococcus* spp. strains—catalytically active towards complex hydrophobic organic compounds—from the Regional Specialised Collection of Alkanotrophic Microorganisms (acronym IEGM, Perm, Russia; World Federation for Culture Collections # 285, http://www.iegmcol.ru/strains, accessed on 6 April 2022) [38]. Previously, the strains were taxonomically affiliated based on their phenotypic and chemotaxonomic properties and were represented by *R. erythropolis* (3 strains), *R*. *fascians* (2 strains), *R*. *jostii* (1 strain), *R*. *opacus* (1 strain), *R*. *qingshengii* (3 strains), *R. rhodochrous* (20 strains), and *R*. *ruber* (10 strains) (Table S3).

### 3.2. Reagents and Nanomaterials

All commercially available compounds and solvents were of reagent grade and were used without further treatment unless otherwise specified. (–)-Isopulegol (C_10_H_18_O, (1*R*,2*S*,5*R*)-5-methyl-2-(prop-1-en-2-yl)cyclohexan-1-ol, CAS No. 89-79-2, ${\left[\alpha \right]}_{D}^{31}$ = −21 (c = 0.4, CHCl_3_)) was purchased from Sigma-Aldrich (St. Louis, MO, USA). Samples of HNTs (outer diameter, ~50 nm, inner diameter, ~15 nm, and length of 700 nm to 2 μm) were purchased from Sigma-Aldrich (St. Louis, MO, USA).

### 3.3. Cultivation Conditions

*Rhodococcus* cultures were grown on solid (1.5% *w*/*v* agar) media and in liquid media based on (1) nutrient BTN broth (Biotechnovatsiya LLC, Elektrogorsk, Russia) and (2) mineral salt medium RS containing (g/L): K_2_HPO_4_–2.0; KH_2_PO_4_–2.0; KNO_3_–1.0; (NH_4_)_2_SO_4_–2.0; NaCl–1.0; MgSO_4_–0.2; CaCl_2_–0.02, FeCl_3_ × 7H_2_O–0.01, and trace element solutions according to Postgate (0.1% *v*/*v*) [39]. The media were supplemented with (–)-isopulegol to a final concentration of 0.025% *v*/*v* [18], a suspension of HNTs (0.1 g/L), or yeast extract (0.1 g/L) (FBIS SRCAMB, Obolensk, Russia), either individually or in combination. Solutions of yeast extract and trace elements were sterilized separately and added to the pre-autoclaved RS medium base.

*Rhodococcus* cells harvested from BTN agar slants were streaked on Petri dishes containing agar medium (with/without additives). RS-based liquid media (20 mL and 100 mL in 100 and 250 mL flasks, respectively) were inoculated with *Rhodococcus* cultures grown on BTN broth (2 days, 28 °C) to the initial optical density of OD_600_ 0.2. In biotransformation experiments, the controls were: (i) sterile cell-free RS medium with (–)-isopulegol (0.025% *v*/*v*) (abiotic control) and (ii) a culture of rhodococci grown in a mineral medium without (–)-isopulegol and/or HNTs (for differentiation of metabolites). Flasks with experimental and control variants were incubated on an orbital shaker Certomat IS (Sartorius, Göttingen, Germany,160 rpm) at 28 °C for 5 days with periodic sampling for analysis. The inoculated dishes were incubated for 2–10 days depending on the intensity of growth on various media.

### 3.4. Phase-Contrast and Fluorescent Microscopy

Samples of cell suspensions were screened in an Axio Imager M2 optical microscope (Carl Zeiss Microscopy GmbH, Jena, Germany) equipped with an Axiocam 506 Color camera or in an Olympus FV1000 confocal laser scanning microscope (Olympus Corporation, Tokyo, Japan). Cell suspensions were stained with a fluorescent dye Live/Dead^®^ Bac*Light*^TM^ Bacterial Viability Kit (Invitrogen, Carlsbad, CA, USA) to differentiate living and dead cells in accordance with the protocol for sample preparation and viewing mode, as recommended by the manufacturer. Cell suspensions were also stained with Nile Red dye (Nanjing Dubai Biotechnology Co., Nanjing, China) to detect intracellular lipids [40]. Details of procedures used in the study are given in Supplementary Materials.

### 3.5. Atomic Force and Confocal Laser Scanning Microscopy (AFM-CLSM)

Bacterial cell morphology was analyzed using a combined scanning system consisting of an Olympus FV 1000 confocal laser scanning microscope (CLSM) (Olympus Corporation, Tokyo, Japan) and an Asylum MFP-3D atomic force microscope (AFM) (Asylum Research, Santa Barbara, CA, USA). AFM scanning was performed in a semi-contact mode in air using an AC240TS silicon cantilever with resonance frequency of 50–90 kHz and contact stiffness of 0.5–4.4 N/m. Images were analyzed using the FV10-ASW 3.1 program (Olympus Corporation, Tokyo, Japan). To obtain the combined AFM-CLSM images, the CLSM image was imported into the AFM software (Igor Pro 6/22A Wave Metrics, Portland, OR, USA).

### 3.6. Transmission Electron Microscopy (TEM)

Cells were harvested from the cultures grown for 72 h on solid media, fixed in 2.5% glutaraldehyde (*w*/*v*) in 0.1 M sodium cacodylate buffer (pH 7.2) for 2.5 h, and then post-fixed in 1% (*w*/*v*) osmium tetroxide in the same buffer. The fixed material was dehydrated through a series of ethanol solutions, including absolute ethanol saturated with uranyl acetate, and embedded in araldite. Thin sections were prepared on an 8800 Ultrotome III (LKB-Produkter, Stockholm, Sweden) and stained with lead citrate. Ultrathin sections were examined in a JEM-1400 electron microscope (Jeol, Tokyo, Japan).

### 3.7. Energy Dispersive X-ray Spectroscopy (EDX) with Elemental Mapping

Suspensions of harvested cells in water (without any fixative) were applied onto Formvar-coated and carbon-reinforced copper grids and air-dried. TEM-EDX with elemental mapping were performed using a JEM-1400 microscope (Jeol, Tokyo, Japan) equipped with an energy dispersive X-ray analysis system (EDXA, Inca Energy-350, Oxford Instruments, Abingdon, UK), operating at accelerating voltage of 80 keV (tilt angle, 15°). EDX spectra and elemental maps were obtained using AZtec software (Oxford Instruments, Abingdon, UK).

### 3.8. Zeta Potential Measurement

Zeta potential of bacterial cells was estimated by the dynamic light scattering technique using a ZetaSizer Nano ZS (Malvern Instruments, Malvern, UK) and the Malvern ZetaSizer software, v. 2.2 (Malvern Instruments, Malvern, UK). Cells were washed twice and resuspended in 0.1 M KNO_3_ (pH 7.0) to OD_600_ of 2.0. Measurements were carried out in a U-shaped cuvette with gold-plated electrodes at 25 °C (pH 7.0).

### 3.9. Metabolic Activity Measurements

To assess the abundance of metabolically active cells, the cell suspension (100 µL per well) and a 0.2% aqueous solution of iodonitrotetrazolium chloride (50 µL per well) were added to microplates and incubated at 28 °C for 2 h. The optical density (λ = 630 nm) of formazane was recorded on a Multiskan Ascent microplate spectrophotometer (Thermo Electron Corporation, Waltham, MA, USA).

The rates of O_2_ consumption and CO_2_ release by growing *Rhodococcus* cultures (100 mL per 300 mL vial) were recorded using a 10-channel Columbus Micro-Oxymax respirometer (Columbus Instruments, Columbus, OH, USA) with stirring (300 rpm, 28 ± 2 °C) on an Ikamag^®^ RO10 power magnetic stirrer (IKA-Werke, Staufen, Germany).

### 3.10. Extraction and Analysis of Residual (–)-Isopulegol and Its Derivatives

To extract the residual (–)-isopulegol and its derivatives, an equivalent volume of ethyl acetate was added to the cell suspension acidified with 10% HCl solution; after separation of the resulting emulsion, the ethyl acetate fraction was taken. Ethyl acetate was added to the remaining aqueous fraction for re-extraction; the procedure for separation and selection of fractions was repeated twice. The ethyl acetate fractions were combined and evaporated on a rotary evaporator Laborota 4000 (Heidolph, Schwabach, Germany); the residue was successively washed with a 1% NaHCO_3_ aqueous solution and distilled water (up to pH 7.0) and dried over anhydrous Na_2_SO_4_. The washed residue was used for analyses.

Qualitative analysis was carried out by thin layer chromatography (TLC) on Alugram^®^ Xtra SIL G/UV254 plates (Macherey-Nagel, Düren, Germany) in the *n*-hexane-ethyl acetate (1:1) system. Separated components were detected after drying and keeping the plates in iodine vapor.

Column chromatography (CC): silica gel (SiO_2_; 60–200μm; Macherey-Nagel), hexane:EtOAc from 100:0 to 0:100 as eluent.

GC-MS (products analysis): Agilent 7890A with a quadrupole mass spectrometer Agilent 5975C as a detector, HP-5MS quartz column, 30,000 × 0.25 mm, He (1 atm) as carrier gas. The column temperature was programmed from 50 to 280 °C. MS were recorded in the range of 50–700 *m/z* and compared with those from the NIST08 Library.

HR-MS: DFS-Thermo-Scientific spectrometer in a full scan mode (15–500 *m/z*, 70 eV electron-impact ionization, direct sample introduction).

Optical rotation: polAAr 3005 spectrometer (Index Instruments, Huntingdon, UK), CHCl_3_ solution.

^1^H and ^13^C NMR: Avance-III 400 apparatus (Bruker, Billerica, MA, USA) at 400.13 MHz (^1^H) and 100.61 MHz (^13^C) in CDCl_3_; chemical shifts δ in ppm rel. to residual CHCl_3_ (δ(H) 7.24, δ (C) 76.90 ppm), *J* in Hz; structure determinations by analyzing the ^1^H NMR spectra, including ^1^H-^1^H 2D homonuclear correlation (COSY, NOESY); *J*-modulated ^13^C NMR spectra (JMOD), and ^13^C-^1^H 2D heteronuclear correlation with one-bond and long-range spin-spin coupling constants (HSQC, ^1^*J*(C,H) = 145 Hz; HMBC, ^2,3^*J*(C,H) = 7 Hz).

Biotransformation of (−)-isopulegol (0.182 g) by *R. rhodochrous* IEGM 1362 without HNTs (or in the presence of HNTs) in RS medium (100 mL) for 5 days proceeded with the formation of compounds **2** and **3**. To isolate (−)-isopulegol metabolites, the culture media were acidified with 10% HCl solution and extracted three times with an equal volume of ethyl acetate. The combined organic layers were washed with an aqueous solution of 1% NaHCO_3_ and then with distilled water to a final pH of 7.0. The ethyl acetate extract was dried over anhydrous Na_2_SO_4_, filtered and concentrated. The reaction mixture was separated on an SiO_2_ column (hexane:EtOAc from 100:0 to 0:100 as eluent). The biotransformation products of (−)-isopulegol were analyzed using GC–MS. According to GC–MS, conversion of (−)-isopulegol was 90.2%, selectivity towards compound **3** (*m/z* 184) was 25.7%; and for compound **2** (*m/z* 170), selectivity was 66.9%. Products **2** (0.017 g, 8.5%) and **3** (0.028 g, 12.9%) were isolated by column chromatography on SiO_2_.

(1*R*,2*S*,5*R*)-5-(Hydroxymethyl)-2-(prop-1-en-2-yl)cyclohexanol (**2**). White powder, mp 67.8 °C, ${\left[\alpha \right]}_{580}^{26}$ = −.5 (c = 5, CHCl_3_). NMR ^1^H (400 MHz, CDCl_3_, δ, ppm, J/Hz): 0.90–1.05 (m, 2H, 2-H_a_, 6-H), 1.26–1.39 (m, 1H, 5-H_a_), 1.55–1.68 (m, 1H, 1-H_a_), 1.68–1.70 (m, 3H, 9-Me), 1.69–1.82 (m, 2H, 5-H_e_, 6-H’), 1.86–1.95 (m, 1H, 4-H_a_), 2.11 (dm, 1H, ^2^*J* 12.2, 2-H_e_), 3.43–3.54 (m, 3H, 3-H_a_, 10-H, 10-H’), 4.82–4.86 (m, 1H, 8-H), 4.87–4.91 (m, 1H, 8-H’). NMR ^13^C (100 MHz, CDCl_3_, δ, ppm): 39.1 (d, C-1), 36.8 (t, C-2), 70.1 (d,C-3), 54.3 (d, C-4), 29.0 (t,C-5), 28.5 (t,C-6), 146.2 (s, C-7), 113.0 (t, C-8), 19.1 (q, C-9), 67.8 (t, C-10). HR-MS: *m/z* calcd. for C_10_H_18_O_2_: 170.1301. Found: 170.1299.

(1*R*,3*R*,4*S*)-3-Hydroxy-4-(prop-1-en-2-yl)cyclohexanecarboxylic acid (**3**). White powder, mp 121.2–121.9 °C, ${\left[\alpha \right]}_{580}^{26}$ = −9.1 (c = 0.4, CHCl_3_). NMR ^1^H (400 MHz, CDCl_3_, δ, ppm): 1.28–1.51 (m, 3H, 2-H_a_, 5-H_a_, 6-H), 1.69 (br.s, 3H, 9-Me), 1.72–1.82 (m, 1H, 5-H_e_), 1.90–1.97 (m, 1H, 4-H_a_), 1.95–2.04 (m, 1H, 6-H’), 2.30–2.47 (m, 2H, 1-H_a_, 2-H_e_), 3.44–3.55 (m, 1H, 3-H_a_), 4.84–4.87 (m, 1H, 8-H), 4.89–4.92 (m, 1H, 8-H’). NMR ^13^C (100 MHz, CDCl_3_, δ, ppm): 41.6 (d, C-1), 35.6 (t, C-2), 69.6 (d,C-3), 53.4 (d, C-4), 28.7 (t,C-5), 27.9 (t,C-6), 145.6 (s, C-7), 113.5 (t, C-8), 19.0 (q, C-9), 180.5 (s, C-10). HR-MS: *m/z* calcd. for C_10_H_16_O_3_: 184.1094. Found: 184.1096.

### 3.11. X-ray Data for Compounds 2 and 3

X-ray crystallographic data were obtained on a Kappa Apex II diffractometer (Bruker, Billerica, MA, USA) with a CCD detector using graphite monochromated MoKa radiation (λ = 0.71073 Å). Experimental data reduction was performed using APEX2 suite (Bruker AXS Inc. APEX2 (Version 2.0), SAINT (Version 8.18c), and SADABS (Version 2.11); Bruker Advanced X-ray Solutions, Madison, WI, USA, 2000–2012). The structures were solved by direct methods and refined by the full-matrix least-squares technique against F^2^ in the anisotropic-isotropic approximation. The H atom positions were calculated with the riding model. All calculations were performed using SHELXTL-2018/3. CCDC 2168968 (**2**) and 2168969 (**3**) contain the Supplementary Crystallographic Data for this paper. These data can be obtained free of charge from The Cambridge Crystallographic Data Centre via http://www.ccdc.cam.ac.uk/conts/retrieving.html, accessed on 6 April 2022(or from the CCDC, 12 Union Road, Cambridge CB2 1EZ, UK; Fax: +44-1223-336033; E-mail: deposit@ccdc.cam.ac.uk).

Crystal data **2**: C_10_H_18_O_2_, M = 170.24, monoclinic, space group P2_1_, at 296 K: *a* = 11.5798(8), *b* = 7.1097(4), c = 13.2094(9) Å, β = 109.144(3)°, V = 1027.37(12) Å^3^, Z = 4, d_calc_ = 1.101 g·cm^−3^, µ = 0.074 mm^−1^, a total of 11262 (θ_max_ = 25.74◦), 3938 unique (R_int_ = 0.0571), 2829 [I > 2σ(I)], 223 parameters. GooF = 0.947, R_1_ = 0.0426, wR_2_ = 0.0996 [I > 2σ(I)], R_1_ = 0.0724, wR_2_ = 0.1163 (all data), max/min diff. peak 0.12/-0.13 e·Å^−3^.

Crystal data **3**: C_10_H_16_O_3_, M = 184.23, monoclinic, space group P2_1_, at 296 K: *a* = 5.9661(2), *b* = 11.1272(5), c = 7.8484(4) Å, β = 95.0116(16)◦, V = 519.03(4) Å^3^, Z = 2, d_calc_ = 1.179 g·cm^−3^, µ = 0.086 mm^−1^, a total of 15008 (θ_max_ = 27.61◦), 2411 unique (R_int_ = 0.0409), 2248 [I > 2σ(I)], 127 parameters. GooF = 1.015, R_1_ = 0.0332, wR_2_ = 0.0870 [I > 2σ(I)], R_1_ = 0.0365, wR_2_ = 0.0906 (all data), max/min diff. peak 0.18/-0.12 e·Å^−3^.

### 3.12. In Silico Analysis of (–)-Isopulegol and Its Derivatives

Ecotoxicity and solubility of (–)-isopulegol and its derivatives were calculated using the Ecological Structure Activity Relationship program (ECOSAR, EPA, Washington, DC, USA). Potential acute toxicity and chronic toxicity to aquatic and terrestrial organisms was predicted based on the available data on toxic effects of organic compounds of various chemical classes using a computational analysis of structure–function relationship in molecules.

The estimated biological activity of the obtained (–)-isopulegol derivatives was predicted based on their structural formulas using the PASS software (Prediction of Activity Spectra for Substances, http://www.pharmaexpert.ru/passonline/index.php, accessed on 6 April 2022, [41]). Exploring the biological potential of substances generated a list of anticipated types of biological activity, including the evaluation of detection (P_a_)/non-detection (P_i_) probabilities of the latter. The highest probability of biological activity was taken as 1.

## 4. Conclusions

*R. rhodochrous* was shown for the first time to biotransform (–)-isopulegol with the formation of previously unidentified compounds (1*R*,2*S*,5*R*)-5-(hydroxymethyl)-2-(prop-1-en-2-yl)cyclohexanol (2) and (1*R*,3*R*,4*S*)-3-hydroxy-4-(prop-1-en-2-yl)cyclohexanecarboxylic acid (3). These compounds were deposited in the CCDC database. According to in silico analysis, the compounds obtained in this work may have greater solubility in water and less ecotoxicity compared to the parent monoterpenoid. The estimated biological activities of metabolites showed that both compounds may very likely have antitumor activity and be used as respiratory analeptics and prevention agents of cancer of the genitourinary system. The obtained data dictate the prospects for further study of compounds, including assessment of their in vitro and in vivo bioactivity.

A comprehensive study of *Rhodococcus* responses in the presence of (–)-isopulegol, HNTs, and their combination showed that, in the concentrations used, neither individual nor combined action markedly affected the morphology and vital processes of bacterial cells, indicating their high stability and the need for future studies with elevated concentrations of these substances.

## Figures and Tables

**Table 1:** Changes in ethyl acetate extract (%) composition during biotransformation of (–)-isopulegol by *R*. *rhodochrous* IEGM 1362.

Culture Conditions	Days	(–)-Isopulegol (*m/z* 154, 7.84 min)	Compound 2 (*m/z* 170, 10.66 min)	Compound 3 (*m/z* 184, 11.50 min)
Medium, 25 mL				
(–)-Isopulegol	2	15.2	84.8	−
	3	45.7	−	54.3
	5	19.4	−	80.6
(–)-Isopulegol and HNTs	2	40.8	59.2	−
	3	−	100	−
	5	12.0	72.2	15.7
Medium, 100 mL				
(–)-Isopulegol	2	−	−	100
(–)-Isopulegol and HNTs	2	−	−	100

**Table 2:** Estimated ecotoxicity and solubility of (–)-isopulegol and its derivatives.

Test Organisms (Index, Exposure)	Concentration, mg/L		
	(–)-Isopulegol	Compound 2	Compound 3
Water solubility at 25 °C	308.6	345.2	6303.00
ECOSAR Class	Neutral Organics	Neutral Organics	Neutral Organics-acid
Acute toxicity			
Fish (LD_50_, 96 h)	7.39	72.75	1138.44
Daphnia (LD_50_, 48 h)	4.76	42.53	654.68
Green algae (ED_50_, 96 h)	5.99	35.74	513.99
Chronic toxicity			
Fish (ED_50_, 30 d)	0.84	7.36	112.96
Daphnia (ED_50_, 21 d)	0.66	4.49	66.15
Green algae (ED_50_, 16 d)	2.08	9.98	138.47

**Table 3:** Estimated biological activity of (–)-isopulegol and its derivatives.

Estimated Activity	(–)-Isopulegol		Compound 2		Compound 3	
	P_a_	P_i_	P_a_	P_i_	P_a_	P_i_
Carminative	0.976	0.000	0.930	0.001	0.928	0.001
Anti–eczema	0.929	0.004	0.918	0.004	0.908	0.004
Neuromuscular acetyl choline blocker	0.751	0.003	0.677	0.005	0.682	0.005
Inhibitor of β-glucuronidase	–	–	0.670	0.005	0.714	0.004
Immunosuppressive	0.755	0.010	0.720	0.014	0.731	0.013
Stimulator of NFκB transcription factor	0.747	0.003	0.730	0.004	0.730	0.004
Inhibitor of retinol dehydrogenase	0.738	0.002	0.739	0.002	0.693	0.002
Respiratory analeptic	–	–	0.568	0.026	0.686	0.016
Anti-inflammatory	0.690	0.024	0.645	0.024	0.692	0.013
Antitumor	–	–	0.550	0.057	0.482	0.002

## Data Availability

Data are contained within the article and Supplementary Materials.

## Supplementary Materials

The following supporting information can be downloaded at: https://www.mdpi.com/article/10.3390/ph15080964/s1, Table S1: Morphometric parameters of R. rhodochrous IEGM 1362 cells; Table S2: Parameters of the cell surface of R. rhodochrous IEGM 1362; Table S3: Rhodococcus strains used in the research; File S1: The crystallographic data of compounds 2 (CCDC 2168968); File S2: The crystallographic data of compounds 3 (CCDC 2168969).

## Abbreviations

AFM	atomic force microscopy
CCDC	the Cambridge Crystallographic Data Center
CLSM	confocal laser scanning microscopy
EDX	energy dispersive X-ray spectrometry
GC-MS	gas chromatography-mass spectrometry
TEM	transmission electron microscopy
TLC	thin layer chromatography
HMBC	heteronuclear multiple bond correlation
HNTs	halloysite nanotubes
HR-MS	high resolution mass spectrometry
NMR	nuclear magnetic resonance
OD	optical density
