# Supplementary material for: Biotransformation of (–)-Isopulegol by Rhodococcus rhodochrous
Source: Pharmaceuticals (Basel). 2022 Aug 3;15(8):964. doi: 10.3390/ph15080964 (PMC9412403; doi:10.3390/ph15080964)
Supplement: Supplementary file 1 [file pharmaceuticals-15-00964-s001.zip › pharmaceuticals-1821943-supplementary/CCDC 2168968 (I10_144_1nh).doc.pdf]

```
data_I10_1441

_audit_creation_method            'SHELXL-2018/3'
_shelx_SHELXL_version_number      '2018/3'
_chemical_name_systematic         ?
_chemical_name_common             ?
_chemical_melting_point           ?
_chemical_formula_moiety          ?
_chemical_formula_sum
 'C10 H18 O2'
_chemical_formula_weight          170.24

loop_
 _atom_type_symbol
 _atom_type_description
 _atom_type_scat_dispersion_real
 _atom_type_scat_dispersion_imag
 _atom_type_scat_source
 'C'  'C'   0.0033   0.0016
 'International Tables Vol C Tables 4.2.6.8 and 6.1.1.4'
 'H'  'H'   0.0000   0.0000
 'International Tables Vol C Tables 4.2.6.8 and 6.1.1.4'
 'O'  'O'   0.0106   0.0060
 'International Tables Vol C Tables 4.2.6.8 and 6.1.1.4'

_space_group_crystal_system       monoclinic
_space_group_IT_number            4
_space_group_name_H-M_alt         'P 21'
_space_group_name_Hall            'P 2yb'

_shelx_space_group_comment
;
The symmetry employed for this shelxl refinement is uniquely defined
by the following loop, which should always be used as a source of
symmetry information in preference to the above space-group names.
They are only intended as comments.
;

loop_
 _space_group_symop_operation_xyz
 'x, y, z'
 '-x, y+1/2, -z'
```

```
_cell_length_a                    11.5798(8)
_cell_length_b                    7.1097(4)
_cell_length_c                    13.2094(9)
_cell_angle_alpha                 90
_cell_angle_beta                  109.144(3)
_cell_angle_gamma                 90
_cell_volume                      1027.37(12)
_cell_formula_units_Z             4
_cell_measurement_temperature     299(2)
_cell_measurement_reflns_used     ?
_cell_measurement_theta_min       ?
_cell_measurement_theta_max       ?

_exptl_crystal_description        ?
_exptl_crystal_colour             ?
_exptl_crystal_density_meas       ?
_exptl_crystal_density_method     ?
_exptl_crystal_density_diffrn     1.101
_exptl_crystal_F_000              376
_exptl_transmission_factor_min    ?
_exptl_transmission_factor_max    ?
_exptl_crystal_size_max           0.640
_exptl_crystal_size_mid           0.160
_exptl_crystal_size_min           0.110
_exptl_absorpt_coefficient_mu     0.074
_shelx_estimated_absorpt_T_min    0.954
_shelx_estimated_absorpt_T_max    0.992
_exptl_absorpt_correction_type    ?
_exptl_absorpt_correction_T_min   ?
_exptl_absorpt_correction_T_max   ?
_exptl_absorpt_process_details    ?
_exptl_absorpt_special_details    ?
_diffrn_ambient_temperature       299(2)
_diffrn_radiation_wavelength      0.71073
_diffrn_radiation_type            MoK\a
_diffrn_source                    ?
_diffrn_measurement_device_type   ?
_diffrn_measurement_method        ?
_diffrn_detector_area_resol_mean  ?
_diffrn_reflns_number             11262
_diffrn_reflns_av_unetI/netI      0.0625
_diffrn_reflns_av_R_equivalents   0.0571
_diffrn_reflns_limit_h_min        -14
_diffrn_reflns_limit_h_max        14
```

```
_diffrn_reflns_limit_k_min        -8
_diffrn_reflns_limit_k_max        8
_diffrn_reflns_limit_l_min        -15
_diffrn_reflns_limit_l_max        16
_diffrn_reflns_theta_min          2.034
_diffrn_reflns_theta_max          25.743
_diffrn_reflns_theta_full         25.242
_diffrn_measured_fraction_theta_max   0.998
_diffrn_measured_fraction_theta_full  0.999
_diffrn_reflns_Laue_measured_fraction_max    0.998
_diffrn_reflns_Laue_measured_fraction_full   0.999
_diffrn_reflns_point_group_measured_fraction_max   0.997
_diffrn_reflns_point_group_measured_fraction_full  0.999
_reflns_number_total              3938
_reflns_number_gt                 2829
_reflns_threshold_expression      'I > 2\s(I)'
_reflns_Friedel_coverage          0.839
_reflns_Friedel_fraction_max      0.996
_reflns_Friedel_fraction_full     1.000

_reflns_special_details
;
 Reflections were merged by SHELXL according to the crystal
 class for the calculation of statistics and refinement.

 _reflns_Friedel_fraction is defined as the number of unique
 Friedel pairs measured divided by the number that would be
 possible theoretically, ignoring centric projections and
 systematic absences.
;

_computing_data_collection        ?
_computing_cell_refinement        ?
_computing_data_reduction         ?
_computing_structure_solution     ?
_computing_structure_refinement 'SHELXL-2018/3 (Sheldrick, 2018)'
_computing_molecular_graphics     ?
_computing_publication_material   ?
_refine_special_details           ?
_refine_ls_structure_factor_coef  Fsqd
_refine_ls_matrix_type            full
_refine_ls_weighting_scheme       calc
_refine_ls_weighting_details
'w=1/[\s^2^(Fo^2^)+(0.0614P)^2^+0.0241P] where P=(Fo^2^+2Fc^2^)/3'
```

```
_atom_sites_solution_primary      ?
_atom_sites_solution_secondary    ?
_atom_sites_solution_hydrogens    geom
_refine_ls_hydrogen_treatment     constr
_refine_ls_extinction_method      none
_refine_ls_extinction_coef        .
_refine_ls_abs_structure_details
;
 Flack x determined using 1011 quotients [(I+)-(I-)]/[(I+)+(I-)]
 (Parsons, Flack and Wagner, Acta Cryst. B69 (2013) 249-259).
;
_refine_ls_abs_structure_Flack    0.0(7)
_chemical_absolute_configuration  ?
_refine_ls_number_reflns          3938
_refine_ls_number_parameters      223
_refine_ls_number_restraints      1
_refine_ls_R_factor_all           0.0724
_refine_ls_R_factor_gt            0.0426
_refine_ls_wR_factor_ref          0.1163
_refine_ls_wR_factor_gt           0.0996
_refine_ls_goodness_of_fit_ref    0.947
_refine_ls_restrained_S_all       0.947
_refine_ls_shift/su_max           0.000
_refine_ls_shift/su_mean          0.000

loop_
 _atom_site_label
 _atom_site_type_symbol
 _atom_site_fract_x
 _atom_site_fract_y
 _atom_site_fract_z
 _atom_site_U_iso_or_equiv
 _atom_site_adp_type
 _atom_site_occupancy
 _atom_site_site_symmetry_order
 _atom_site_calc_flag
 _atom_site_refinement_flags_posn
 _atom_site_refinement_flags_adp
 _atom_site_refinement_flags_occupancy
 _atom_site_disorder_assembly
 _atom_site_disorder_group
O1 O 0.75563(19) -0.2637(3) 0.97961(16) 0.0497(5) Uani 1 1 d . . . . .
H1 H 0.774804 -0.258928 1.045080 0.075 Uiso 1 1 calc R U . . .
C1 C 0.7634(2) -0.0792(3) 0.9384(2) 0.0378(6) Uani 1 1 d . . . . .
```

H1A H 0.837170 -0.018062 0.985948 0.045 Uiso 1 1 calc R U . . .
O2 O 0.5517(2) 0.5377(3) 0.8601(2) 0.0709(7) Uani 1 1 d . . . . .
H2 H 0.611515 0.591833 0.900775 0.106 Uiso 1 1 calc R U . . .
C2 C 0.7765(3) -0.0974(4) 0.8280(2) 0.0439(7) Uani 1 1 d . . . . .
H2A H 0.703715 -0.162783 0.781859 0.053 Uiso 1 1 calc R U . . .
C3 C 0.7777(3) 0.0994(5) 0.7810(3) 0.0571(9) Uani 1 1 d . . . . .
H3A H 0.851229 0.164876 0.823241 0.069 Uiso 1 1 calc R U . . .
H3B H 0.780051 0.087604 0.708505 0.069 Uiso 1 1 calc R U . . .
C4 C 0.6663(3) 0.2156(5) 0.7790(3) 0.0589(9) Uani 1 1 d . . . . .
H4A H 0.672056 0.339715 0.750537 0.071 Uiso 1 1 calc R U . . .
H4B H 0.592847 0.155754 0.732358 0.071 Uiso 1 1 calc R U . . .
C5 C 0.6578(3) 0.2342(4) 0.8907(2) 0.0445(7) Uani 1 1 d . . . . .
H5A H 0.732061 0.297192 0.935919 0.053 Uiso 1 1 calc R U . . .
C6 C 0.6541(3) 0.0387(4) 0.9367(2) 0.0435(7) Uani 1 1 d . . . . .
H6A H 0.651735 0.050558 1.009184 0.052 Uiso 1 1 calc R U . . .
H6B H 0.579961 -0.024743 0.894113 0.052 Uiso 1 1 calc R U . . .
C7 C 0.8868(3) -0.2111(4) 0.8284(3) 0.0543(8) Uani 1 1 d . . . . .
C8 C 0.8753(4) -0.3469(6) 0.7559(4) 0.0973(15) Uani 1 1 d . . . . .
H8A H 0.943575 -0.415127 0.755167 0.117 Uiso 1 1 calc R U . . .
H8B H 0.799138 -0.373294 0.706199 0.117 Uiso 1 1 calc R U . . .
C9 C 1.0056(3) -0.1663(6) 0.9077(3) 0.0795(12) Uani 1 1 d . . . . .
H9A H 1.068428 -0.237328 0.892388 0.119 Uiso 1 1 calc R U . . .
H9B H 1.004485 -0.197809 0.978063 0.119 Uiso 1 1 calc R U . . .
H9C H 1.021874 -0.034340 0.904729 0.119 Uiso 1 1 calc R U . . .
C10 C 0.5493(3) 0.3478(4) 0.8949(3) 0.0613(9) Uani 1 1 d . . . . .
H10A H 0.474886 0.287817 0.850056 0.074 Uiso 1 1 calc R U . . .
H10B H 0.547687 0.348247 0.967870 0.074 Uiso 1 1 calc R U . . .
O3 O 0.31876(18) 0.6517(3) 0.78431(18) 0.0538(6) Uani 1 1 d . . . . .
H3 H 0.391833 0.626356 0.807492 0.081 Uiso 1 1 calc R U . . .
O4 O 0.1727(2) 1.3508(3) 0.80714(18) 0.0602(6) Uani 1 1 d . . . . .
H4 H 0.214251 1.437203 0.796521 0.090 Uiso 1 1 calc R U . . .
C11 C 0.3002(3) 0.8126(4) 0.7150(2) 0.0408(7) Uani 1 1 d . . . . .
H11 H 0.379565 0.871976 0.724896 0.049 Uiso 1 1 calc R U . . .
C12 C 0.2458(2) 0.7494(4) 0.5987(2) 0.0423(7) Uani 1 1 d . . . . .
H12 H 0.171554 0.677855 0.592915 0.051 Uiso 1 1 calc R U . . .
C13 C 0.2064(3) 0.9206(5) 0.5238(2) 0.0514(8) Uani 1 1 d . . . . .
H13A H 0.278453 0.989704 0.523298 0.062 Uiso 1 1 calc R U . . .
H13B H 0.164339 0.877487 0.451377 0.062 Uiso 1 1 calc R U . . .
C14 C 0.1228(3) 1.0505(4) 0.5590(2) 0.0529(8) Uani 1 1 d . . . . .
H14A H 0.048558 0.983874 0.555573 0.063 Uiso 1 1 calc R U . . .
H14B H 0.100519 1.157170 0.510712 0.063 Uiso 1 1 calc R U . . .
C15 C 0.1856(2) 1.1203(4) 0.6731(2) 0.0431(7) Uani 1 1 d . . . . .
H15 H 0.261635 1.182886 0.674721 0.052 Uiso 1 1 calc R U . . .
C16 C 0.2199(3) 0.9507(4) 0.7475(2) 0.0454(7) Uani 1 1 d . . . . .

```
H16A H 0.262178 0.993761 0.819914 0.054 Uiso 1 1 calc R U . . .
H16B H 0.145770 0.887197 0.747694 0.054 Uiso 1 1 calc R U . . .
C17 C 0.3284(3) 0.6211(5) 0.5622(3) 0.0518(8) Uani 1 1 d . . . . .
C18 C 0.2871(4) 0.4649(5) 0.5119(4) 0.0816(12) Uani 1 1 d . . . . .
H18A H 0.338138 0.389436 0.487525 0.098 Uiso 1 1 calc R U . . .
H18B H 0.206623 0.428775 0.500310 0.098 Uiso 1 1 calc R U . . .
C19 C 0.4581(3) 0.6824(6) 0.5818(4) 0.0870(13) Uani 1 1 d . . . . .
H19A H 0.495807 0.600457 0.544037 0.130 Uiso 1 1 calc R U . . .
H19B H 0.502615 0.676868 0.657176 0.130 Uiso 1 1 calc R U . . .
H19C H 0.458818 0.809010 0.556775 0.130 Uiso 1 1 calc R U . . .
C20 C 0.1093(3) 1.2616(5) 0.7084(3) 0.0541(8) Uani 1 1 d . . . . .
H20A H 0.080922 1.357003 0.653350 0.065 Uiso 1 1 calc R U . . .
H20B H 0.037996 1.198301 0.715170 0.065 Uiso 1 1 calc R U . . .

loop_
 _atom_site_aniso_label
 _atom_site_aniso_U_11
 _atom_site_aniso_U_22
 _atom_site_aniso_U_33
 _atom_site_aniso_U_23
 _atom_site_aniso_U_13
 _atom_site_aniso_U_12
O1 0.0683(13) 0.0333(10) 0.0479(12) 0.0057(9) 0.0195(11) 0.0004(10)
C1 0.0437(15) 0.0293(13) 0.0390(16) 0.0030(12) 0.0118(13) -0.0009(12)
O2 0.0519(14) 0.0398(11) 0.106(2) 0.0099(13) 0.0050(12) 0.0102(10)
C2 0.0490(16) 0.0437(16) 0.0362(16) -0.0011(13) 0.0102(13) 0.0020(13)
C3 0.071(2) 0.057(2) 0.049(2) 0.0157(16) 0.0272(17) 0.0126(16)
C4 0.064(2) 0.0511(18) 0.056(2) 0.0181(16) 0.0119(17) 0.0124(16)
C5 0.0410(15) 0.0345(14) 0.0546(19) 0.0032(13) 0.0110(14) 0.0035(12)
C6 0.0468(16) 0.0374(14) 0.0478(18) 0.0020(14) 0.0177(14) -0.0003(13)
C7 0.063(2) 0.052(2) 0.054(2) 0.0080(16) 0.0274(17) 0.0117(15)
C8 0.104(3) 0.087(3) 0.110(4) -0.028(3) 0.048(3) 0.016(3)
C9 0.059(2) 0.100(3) 0.083(3) 0.016(2) 0.027(2) 0.023(2)
C10 0.0494(18) 0.0388(16) 0.094(3) 0.0037(18) 0.0215(19) 0.0079(14)
O3 0.0528(12) 0.0559(12) 0.0499(13) 0.0208(10) 0.0130(11) 0.0091(10)
O4 0.0798(16) 0.0473(12) 0.0534(14) -0.0027(10) 0.0215(12) -0.0008(11)
C11 0.0409(15) 0.0402(15) 0.0393(17) 0.0082(13) 0.0103(13) 0.0001(12)
C12 0.0424(15) 0.0423(14) 0.0392(16) 0.0017(13) 0.0094(13) -0.0047(13)
C13 0.0584(18) 0.0512(17) 0.0370(17) 0.0019(14) 0.0051(14) -0.0003(15)
C14 0.0551(18) 0.0481(16) 0.0426(19) 0.0054(15) -0.0015(14) 0.0046(15)
C15 0.0401(15) 0.0428(15) 0.0404(17) 0.0017(13) 0.0051(13) -0.0002(13)
C16 0.0485(16) 0.0495(16) 0.0370(16) 0.0044(14) 0.0123(13) 0.0046(14)
C17 0.063(2) 0.0481(17) 0.047(2) 0.0043(15) 0.0223(16) 0.0031(15)
C18 0.096(3) 0.060(2) 0.105(3) -0.019(2) 0.054(3) -0.002(2)
```

```
C19 0.067(2) 0.100(3) 0.107(3) -0.021(3) 0.045(2) -0.001(2)
C20 0.0547(18) 0.0482(16) 0.052(2) 0.0015(16) 0.0081(15) 0.0065(15)

_geom_special_details
;
 All esds (except the esd in the dihedral angle between two l.s. planes)
 are estimated using the full covariance matrix.  The cell esds are taken
 into account individually in the estimation of esds in distances, angles
 and torsion angles; correlations between esds in cell parameters are only
 used  when  they  are  defined  by  crystal  symmetry.   An  approximate
(isotropic)
 treatment of cell esds is used for estimating esds involving l.s. planes.
;

loop_
 _geom_bond_atom_site_label_1
 _geom_bond_atom_site_label_2
 _geom_bond_distance
 _geom_bond_site_symmetry_2
 _geom_bond_publ_flag
O1 C1 1.434(3) . ?
O1 H1 0.8200 . ?
C1 C6 1.512(4) . ?
C1 C2 1.520(4) . ?
C1 H1A 0.9800 . ?
O2 C10 1.430(4) . ?
O2 H2 0.8200 . ?
C2 C7 1.510(4) . ?
C2 C3 1.533(4) . ?
C2 H2A 0.9800 . ?
C3 C4 1.525(4) . ?
C3 H3A 0.9700 . ?
C3 H3B 0.9700 . ?
C4 C5 1.516(4) . ?
C4 H4A 0.9700 . ?
C4 H4B 0.9700 . ?
C5 C10 1.509(4) . ?
C5 C6 1.523(4) . ?
C5 H5A 0.9800 . ?
C6 H6A 0.9700 . ?
C6 H6B 0.9700 . ?
C7 C8 1.336(5) . ?
C7 C9 1.466(5) . ?
C8 H8A 0.9300 . ?
```

```
C8 H8B 0.9300 . ?
C9 H9A 0.9600 . ?
C9 H9B 0.9600 . ?
C9 H9C 0.9600 . ?
C10 H10A 0.9700 . ?
C10 H10B 0.9700 . ?
O3 C11 1.436(3) . ?
O3 H3 0.8200 . ?
O4 C20 1.419(4) . ?
O4 H4 0.8200 . ?
C11 C16 1.509(4) . ?
C11 C12 1.525(4) . ?
C11 H11 0.9800 . ?
C12 C17 1.511(4) . ?
C12 C13 1.540(4) . ?
C12 H12 0.9800 . ?
C13 C14 1.517(4) . ?
C13 H13A 0.9700 . ?
C13 H13B 0.9700 . ?
C14 C15 1.525(4) . ?
C14 H14A 0.9700 . ?
C14 H14B 0.9700 . ?
C15 C20 1.509(4) . ?
C15 C16 1.524(4) . ?
C15 H15 0.9800 . ?
C16 H16A 0.9700 . ?
C16 H16B 0.9700 . ?
C17 C18 1.302(5) . ?
C17 C19 1.503(5) . ?
C18 H18A 0.9300 . ?
C18 H18B 0.9300 . ?
C19 H19A 0.9600 . ?
C19 H19B 0.9600 . ?
C19 H19C 0.9600 . ?
C20 H20A 0.9700 . ?
C20 H20B 0.9700 . ?

loop_
 _geom_angle_atom_site_label_1
 _geom_angle_atom_site_label_2
 _geom_angle_atom_site_label_3
 _geom_angle
 _geom_angle_site_symmetry_1
 _geom_angle_site_symmetry_3
```

```
 _geom_angle_publ_flag
C1 O1 H1 109.5 . . ?
O1 C1 C6 110.8(2) . . ?
O1 C1 C2 108.9(2) . . ?
C6 C1 C2 112.3(2) . . ?
O1 C1 H1A 108.2 . . ?
C6 C1 H1A 108.2 . . ?
C2 C1 H1A 108.2 . . ?
C10 O2 H2 109.5 . . ?
C7 C2 C1 113.1(2) . . ?
C7 C2 C3 111.7(2) . . ?
C1 C2 C3 109.2(2) . . ?
C7 C2 H2A 107.6 . . ?
C1 C2 H2A 107.6 . . ?
C3 C2 H2A 107.6 . . ?
C4 C3 C2 112.4(3) . . ?
C4 C3 H3A 109.1 . . ?
C2 C3 H3A 109.1 . . ?
C4 C3 H3B 109.1 . . ?
C2 C3 H3B 109.1 . . ?
H3A C3 H3B 107.9 . . ?
C5 C4 C3 110.8(3) . . ?
C5 C4 H4A 109.5 . . ?
C3 C4 H4A 109.5 . . ?
C5 C4 H4B 109.5 . . ?
C3 C4 H4B 109.5 . . ?
H4A C4 H4B 108.1 . . ?
C10 C5 C4 113.7(3) . . ?
C10 C5 C6 110.0(2) . . ?
C4 C5 C6 109.2(2) . . ?
C10 C5 H5A 107.9 . . ?
C4 C5 H5A 107.9 . . ?
C6 C5 H5A 107.9 . . ?
C1 C6 C5 112.3(2) . . ?
C1 C6 H6A 109.1 . . ?
C5 C6 H6A 109.1 . . ?
C1 C6 H6B 109.1 . . ?
C5 C6 H6B 109.1 . . ?
H6A C6 H6B 107.9 . . ?
C8 C7 C9 121.2(3) . . ?
C8 C7 C2 120.0(3) . . ?
C9 C7 C2 118.8(3) . . ?
C7 C8 H8A 120.0 . . ?
C7 C8 H8B 120.0 . . ?
```

H8A C8 H8B 120.0 . . ?
C7 C9 H9A 109.5 . . ?
C7 C9 H9B 109.5 . . ?
H9A C9 H9B 109.5 . . ?
C7 C9 H9C 109.5 . . ?
H9A C9 H9C 109.5 . . ?
H9B C9 H9C 109.5 . . ?
O2 C10 C5 112.9(3) . . ?
O2 C10 H10A 109.0 . . ?
C5 C10 H10A 109.0 . . ?
O2 C10 H10B 109.0 . . ?
C5 C10 H10B 109.0 . . ?
H10A C10 H10B 107.8 . . ?
C11 O3 H3 109.5 . . ?
C20 O4 H4 109.5 . . ?
O3 C11 C16 108.3(2) . . ?
O3 C11 C12 109.5(2) . . ?
C16 C11 C12 112.9(2) . . ?
O3 C11 H11 108.7 . . ?
C16 C11 H11 108.7 . . ?
C12 C11 H11 108.7 . . ?
C17 C12 C11 113.8(2) . . ?
C17 C12 C13 110.9(2) . . ?
C11 C12 C13 110.6(2) . . ?
C17 C12 H12 107.0 . . ?
C11 C12 H12 107.0 . . ?
C13 C12 H12 107.0 . . ?
C14 C13 C12 111.6(2) . . ?
C14 C13 H13A 109.3 . . ?
C12 C13 H13A 109.3 . . ?
C14 C13 H13B 109.3 . . ?
C12 C13 H13B 109.3 . . ?
H13A C13 H13B 108.0 . . ?
C13 C14 C15 110.8(2) . . ?
C13 C14 H14A 109.5 . . ?
C15 C14 H14A 109.5 . . ?
C13 C14 H14B 109.5 . . ?
C15 C14 H14B 109.5 . . ?
H14A C14 H14B 108.1 . . ?
C20 C15 C14 112.6(2) . . ?
C20 C15 C16 112.4(2) . . ?
C14 C15 C16 108.6(2) . . ?
C20 C15 H15 107.7 . . ?
C14 C15 H15 107.7 . . ?

```
C16 C15 H15 107.7 . . ?
C11 C16 C15 112.7(2) . . ?
C11 C16 H16A 109.1 . . ?
C15 C16 H16A 109.1 . . ?
C11 C16 H16B 109.1 . . ?
C15 C16 H16B 109.1 . . ?
H16A C16 H16B 107.8 . . ?
C18 C17 C19 121.0(3) . . ?
C18 C17 C12 120.8(3) . . ?
C19 C17 C12 118.1(3) . . ?
C17 C18 H18A 120.0 . . ?
C17 C18 H18B 120.0 . . ?
H18A C18 H18B 120.0 . . ?
C17 C19 H19A 109.5 . . ?
C17 C19 H19B 109.5 . . ?
H19A C19 H19B 109.5 . . ?
C17 C19 H19C 109.5 . . ?
H19A C19 H19C 109.5 . . ?
H19B C19 H19C 109.5 . . ?
O4 C20 C15 113.8(2) . . ?
O4 C20 H20A 108.8 . . ?
C15 C20 H20A 108.8 . . ?
O4 C20 H20B 108.8 . . ?
C15 C20 H20B 108.8 . . ?
H20A C20 H20B 107.7 . . ?

loop_
 _geom_torsion_atom_site_label_1
 _geom_torsion_atom_site_label_2
 _geom_torsion_atom_site_label_3
 _geom_torsion_atom_site_label_4
 _geom_torsion
 _geom_torsion_site_symmetry_1
 _geom_torsion_site_symmetry_2
 _geom_torsion_site_symmetry_3
 _geom_torsion_site_symmetry_4
 _geom_torsion_publ_flag
O1 C1 C2 C7 -58.1(3) . . . . ?
C6 C1 C2 C7 178.7(2) . . . . ?
O1 C1 C2 C3 176.9(2) . . . . ?
C6 C1 C2 C3 53.7(3) . . . . ?
C7 C2 C3 C4 179.5(3) . . . . ?
C1 C2 C3 C4 -54.7(4) . . . . ?
C2 C3 C4 C5 57.6(4) . . . . ?
```

```
C3 C4 C5 C10 180.0(3) . . . . ?
C3 C4 C5 C6 -56.8(3) . . . . ?
O1 C1 C6 C5 -178.5(2) . . . . ?
C2 C1 C6 C5 -56.4(3) . . . . ?
C10 C5 C6 C1 -177.8(3) . . . . ?
C4 C5 C6 C1 56.7(3) . . . . ?
C1 C2 C7 C8 132.2(3) . . . . ?
C3 C2 C7 C8 -104.2(4) . . . . ?
C1 C2 C7 C9 -49.1(4) . . . . ?
C3 C2 C7 C9 74.5(4) . . . . ?
C4 C5 C10 O2 -61.5(4) . . . . ?
C6 C5 C10 O2 175.7(3) . . . . ?
O3 C11 C12 C17 -63.1(3) . . . . ?
C16 C11 C12 C17 176.2(2) . . . . ?
O3 C11 C12 C13 171.2(2) . . . . ?
C16 C11 C12 C13 50.5(3) . . . . ?
C17 C12 C13 C14 179.7(2) . . . . ?
C11 C12 C13 C14 -53.0(3) . . . . ?
C12 C13 C14 C15 58.6(3) . . . . ?
C13 C14 C15 C20 175.7(3) . . . . ?
C13 C14 C15 C16 -59.2(3) . . . . ?
O3 C11 C16 C15 -175.3(2) . . . . ?
C12 C11 C16 C15 -53.9(3) . . . . ?
C20 C15 C16 C11 -177.8(2) . . . . ?
C14 C15 C16 C11 57.0(3) . . . . ?
C11 C12 C17 C18 131.4(3) . . . . ?
C13 C12 C17 C18 -103.1(4) . . . . ?
C11 C12 C17 C19 -50.9(4) . . . . ?
C13 C12 C17 C19 74.6(4) . . . . ?
C14 C15 C20 O4 -170.6(2) . . . . ?
C16 C15 C20 O4 66.3(3) . . . . ?

_refine_diff_density_max    0.122
_refine_diff_density_min   -0.133
_refine_diff_density_rms    0.030
```
