# Supplementary material for: Biotransformation of (–)-Isopulegol by Rhodococcus rhodochrous
Source: Pharmaceuticals (Basel). 2022 Aug 3;15(8):964. doi: 10.3390/ph15080964 (PMC9412403; doi:10.3390/ph15080964)
Supplement: Supplementary file 1 [file pharmaceuticals-15-00964-s001.zip › pharmaceuticals-1821943-supplementary/CCDC 2168969 (I10_149_1nh).doc.pdf]

```
data_I10_1491

_audit_creation_method            'SHELXL-2018/3'
_shelx_SHELXL_version_number      '2018/3'
_chemical_name_systematic         ?
_chemical_name_common             ?
_chemical_melting_point           ?
_chemical_formula_moiety          ?
_chemical_formula_sum
 'C10 H16 O3'
_chemical_formula_weight          184.23

loop_
 _atom_type_symbol
 _atom_type_description
 _atom_type_scat_dispersion_real
 _atom_type_scat_dispersion_imag
 _atom_type_scat_source
 'C'  'C'   0.0033   0.0016
 'International Tables Vol C Tables 4.2.6.8 and 6.1.1.4'
 'H'  'H'   0.0000   0.0000
 'International Tables Vol C Tables 4.2.6.8 and 6.1.1.4'
 'O'  'O'   0.0106   0.0060
 'International Tables Vol C Tables 4.2.6.8 and 6.1.1.4'

_space_group_crystal_system       monoclinic
_space_group_IT_number            4
_space_group_name_H-M_alt         'P 21'
_space_group_name_Hall            'P 2yb'

_shelx_space_group_comment
;
The symmetry employed for this shelxl refinement is uniquely defined
by the following loop, which should always be used as a source of
symmetry information in preference to the above space-group names.
They are only intended as comments.
;

loop_
 _space_group_symop_operation_xyz
 'x, y, z'
 '-x, y+1/2, -z'
```

```
_cell_length_a                    5.9661(2)
_cell_length_b                    11.1272(5)
_cell_length_c                    7.8484(4)
_cell_angle_alpha                 90
_cell_angle_beta                  95.0116(16)
_cell_angle_gamma                 90
_cell_volume                      519.03(4)
_cell_formula_units_Z             2
_cell_measurement_temperature     296(2)
_cell_measurement_reflns_used     ?
_cell_measurement_theta_min       ?
_cell_measurement_theta_max       ?

_exptl_crystal_description        ?
_exptl_crystal_colour             ?
_exptl_crystal_density_meas       ?
_exptl_crystal_density_method     ?
_exptl_crystal_density_diffrn     1.179
_exptl_crystal_F_000              200
_exptl_transmission_factor_min    ?
_exptl_transmission_factor_max    ?
_exptl_crystal_size_max           0.630
_exptl_crystal_size_mid           0.300
_exptl_crystal_size_min           0.170
_exptl_absorpt_coefficient_mu     0.086
_shelx_estimated_absorpt_T_min    0.948
_shelx_estimated_absorpt_T_max    0.986
_exptl_absorpt_correction_type    ?
_exptl_absorpt_correction_T_min   ?
_exptl_absorpt_correction_T_max   ?
_exptl_absorpt_process_details    ?
_exptl_absorpt_special_details    ?
_diffrn_ambient_temperature       296(2)
_diffrn_radiation_wavelength      0.71073
_diffrn_radiation_type            MoK\a
_diffrn_source                    ?
_diffrn_measurement_device_type   ?
_diffrn_measurement_method        ?
_diffrn_detector_area_resol_mean  ?
_diffrn_reflns_number             15008
_diffrn_reflns_av_unetI/netI      0.0202
_diffrn_reflns_av_R_equivalents   0.0409
_diffrn_reflns_limit_h_min        -7
_diffrn_reflns_limit_h_max        7
```

```
_diffrn_reflns_limit_k_min        -14
_diffrn_reflns_limit_k_max        14
_diffrn_reflns_limit_l_min        -10
_diffrn_reflns_limit_l_max        10
_diffrn_reflns_theta_min          2.605
_diffrn_reflns_theta_max          27.605
_diffrn_reflns_theta_full         25.242
_diffrn_measured_fraction_theta_max   1.000
_diffrn_measured_fraction_theta_full  1.000
_diffrn_reflns_Laue_measured_fraction_max    1.000
_diffrn_reflns_Laue_measured_fraction_full   1.000
_diffrn_reflns_point_group_measured_fraction_max   0.999
_diffrn_reflns_point_group_measured_fraction_full  1.000
_reflns_number_total              2411
_reflns_number_gt                 2248
_reflns_threshold_expression      'I > 2\s(I)'
_reflns_Friedel_coverage          0.900
_reflns_Friedel_fraction_max      0.998
_reflns_Friedel_fraction_full     1.000

_reflns_special_details
;
 Reflections were merged by SHELXL according to the crystal
 class for the calculation of statistics and refinement.

 _reflns_Friedel_fraction is defined as the number of unique
 Friedel pairs measured divided by the number that would be
 possible theoretically, ignoring centric projections and
 systematic absences.
;

_computing_data_collection        ?
_computing_cell_refinement        ?
_computing_data_reduction         ?
_computing_structure_solution     ?
_computing_structure_refinement 'SHELXL-2018/3 (Sheldrick, 2018)'
_computing_molecular_graphics     ?
_computing_publication_material   ?
_refine_special_details           ?
_refine_ls_structure_factor_coef  Fsqd
_refine_ls_matrix_type            full
_refine_ls_weighting_scheme       calc
_refine_ls_weighting_details
'w=1/[\s^2^(Fo^2^)+(0.0453P)^2^+0.0754P] where P=(Fo^2^+2Fc^2^)/3'
```

```
_atom_sites_solution_primary      ?
_atom_sites_solution_secondary    ?
_atom_sites_solution_hydrogens    mixed
_refine_ls_hydrogen_treatment     mixed
_refine_ls_extinction_method      none
_refine_ls_extinction_coef        .
_refine_ls_abs_structure_details
;
 Flack x determined using 1020 quotients [(I+)-(I-)]/[(I+)+(I-)]
 (Parsons, Flack and Wagner, Acta Cryst. B69 (2013) 249-259).
;
_refine_ls_abs_structure_Flack    -0.2(3)
_chemical_absolute_configuration  ?
_refine_ls_number_reflns          2411
_refine_ls_number_parameters      127
_refine_ls_number_restraints      1
_refine_ls_R_factor_all           0.0365
_refine_ls_R_factor_gt            0.0332
_refine_ls_wR_factor_ref          0.0906
_refine_ls_wR_factor_gt           0.0870
_refine_ls_goodness_of_fit_ref    1.015
_refine_ls_restrained_S_all       1.015
_refine_ls_shift/su_max           0.000
_refine_ls_shift/su_mean          0.000

loop_
 _atom_site_label
 _atom_site_type_symbol
 _atom_site_fract_x
 _atom_site_fract_y
 _atom_site_fract_z
 _atom_site_U_iso_or_equiv
 _atom_site_adp_type
 _atom_site_occupancy
 _atom_site_site_symmetry_order
 _atom_site_calc_flag
 _atom_site_refinement_flags_posn
 _atom_site_refinement_flags_adp
 _atom_site_refinement_flags_occupancy
 _atom_site_disorder_assembly
 _atom_site_disorder_group
O1 O 0.3830(2) 0.83793(15) 0.6000(3) 0.0664(5) Uani 1 1 d . . . . .
C1 C 0.3278(3) 0.64203(16) 0.7173(2) 0.0387(4) Uani 1 1 d . . . . .
H1A H 0.190692 0.674158 0.760405 0.046 Uiso 1 1 calc R U . . .
```

```
O2 O 0.6819(2) 0.72803(15) 0.6763(2) 0.0570(4) Uani 1 1 d . . . . .
H2 H 0.753(5) 0.785(3) 0.634(4) 0.062(8) Uiso 1 1 d . . . . .
C2 C 0.2582(3) 0.56462(16) 0.5594(2) 0.0373(4) Uani 1 1 d . . . . .
H2A H 0.173751 0.613513 0.473912 0.045 Uiso 1 1 calc R U . . .
H2B H 0.391927 0.535647 0.510589 0.045 Uiso 1 1 calc R U . . .
O3 O 0.0642(2) 0.38945(13) 0.45101(17) 0.0421(3) Uani 1 1 d . . . . .
H3 H -0.071(5) 0.375(3) 0.436(3) 0.056(7) Uiso 1 1 d . . . . .
C3 C 0.1150(3) 0.45798(15) 0.6048(2) 0.0350(4) Uani 1 1 d . . . . .
H3A H -0.025334 0.487348 0.646008 0.042 Uiso 1 1 calc R U . . .
C4 C 0.2406(3) 0.38072(18) 0.7439(2) 0.0397(4) Uani 1 1 d . . . . .
H4A H 0.381463 0.354826 0.699641 0.048 Uiso 1 1 calc R U . . .
C5 C 0.3043(4) 0.4590(2) 0.9016(3) 0.0508(5) Uani 1 1 d . . . . .
H5A H 0.168332 0.487789 0.947433 0.061 Uiso 1 1 calc R U . . .
H5B H 0.386682 0.410828 0.988951 0.061 Uiso 1 1 calc R U . . .
C6 C 0.4481(4) 0.56613(19) 0.8577(3) 0.0479(5) Uani 1 1 d . . . . .
H6A H 0.481348 0.615083 0.959062 0.057 Uiso 1 1 calc R U . . .
H6B H 0.589535 0.537535 0.820621 0.057 Uiso 1 1 calc R U . . .
C7 C 0.4644(3) 0.74678(17) 0.6608(2) 0.0401(4) Uani 1 1 d . . . . .
C8 C 0.1144(4) 0.2681(2) 0.7866(3) 0.0518(5) Uani 1 1 d . . . . .
C9 C 0.1992(5) 0.1607(2) 0.7609(4) 0.0695(7) Uani 1 1 d . . . . .
H9A H 0.120822 0.092190 0.788437 0.083 Uiso 1 1 calc R U . . .
H9B H 0.337274 0.153639 0.715251 0.083 Uiso 1 1 calc R U . . .
C10 C -0.1066(5) 0.2838(3) 0.8596(5) 0.0901(11) Uani 1 1 d . . . . .
H10A H -0.172754 0.206490 0.875853 0.135 Uiso 1 1 calc R U . . .
H10B H -0.205097 0.330529 0.782208 0.135 Uiso 1 1 calc R U . . .
H10C H -0.084015 0.324574 0.967503 0.135 Uiso 1 1 calc R U . . .

loop_
 _atom_site_aniso_label
 _atom_site_aniso_U_11
 _atom_site_aniso_U_22
 _atom_site_aniso_U_33
 _atom_site_aniso_U_23
 _atom_site_aniso_U_13
 _atom_site_aniso_U_12
O1 0.0331(7) 0.0500(9) 0.1153(16) 0.0227(9) 0.0015(8) 0.0030(7)
C1 0.0300(8) 0.0401(9) 0.0461(10) -0.0031(8) 0.0032(7) -0.0011(7)
O2 0.0287(7) 0.0540(9) 0.0883(12) 0.0204(9) 0.0043(7) 0.0000(6)
C2 0.0329(8) 0.0391(9) 0.0390(8) 0.0038(7) -0.0021(6) -0.0016(7)
O3 0.0267(6) 0.0472(7) 0.0512(8) -0.0073(6) -0.0040(5) -0.0011(6)
C3 0.0256(7) 0.0380(9) 0.0411(9) -0.0001(7) 0.0009(6) 0.0009(6)
C4 0.0310(8) 0.0422(9) 0.0454(10) 0.0069(8) 0.0001(6) -0.0004(8)
C5 0.0558(12) 0.0576(12) 0.0381(10) 0.0080(9) -0.0006(8) -0.0090(10)
C6 0.0470(10) 0.0535(12) 0.0414(10) -0.0006(9) -0.0064(8) -0.0088(9)
```

```
C7 0.0289(8) 0.0403(9) 0.0501(10) -0.0028(8) -0.0011(7) 0.0003(7)
C8 0.0473(11) 0.0506(12) 0.0554(12) 0.0166(9) -0.0082(9) -0.0095(9)
C9 0.0766(17) 0.0500(13) 0.0769(17) 0.0151(12) -0.0219(13) -0.0084(12)
C10 0.0646(17) 0.086(2) 0.123(3) 0.031(2) 0.0290(18) -0.0183(16)

_geom_special_details
;
 All esds (except the esd in the dihedral angle between two l.s. planes)
 are estimated using the full covariance matrix.  The cell esds are taken
 into account individually in the estimation of esds in distances, angles
 and torsion angles; correlations between esds in cell parameters are only
 used when they are defined by crystal symmetry.  An approximate
(isotropic)
 treatment of cell esds is used for estimating esds involving l.s. planes.
;

loop_
 _geom_bond_atom_site_label_1
 _geom_bond_atom_site_label_2
 _geom_bond_distance
 _geom_bond_site_symmetry_2
 _geom_bond_publ_flag
O1 C7 1.205(3) . ?
C1 C7 1.510(3) . ?
C1 C6 1.518(3) . ?
C1 C2 1.536(3) . ?
C1 H1A 0.9800 . ?
O2 C7 1.309(2) . ?
O2 H2 0.85(3) . ?
C2 C3 1.522(2) . ?
C2 H2A 0.9700 . ?
C2 H2B 0.9700 . ?
O3 C3 1.437(2) . ?
O3 H3 0.82(3) . ?
C3 C4 1.533(2) . ?
C3 H3A 0.9800 . ?
C4 C8 1.514(3) . ?
C4 C5 1.534(3) . ?
C4 H4A 0.9800 . ?
C5 C6 1.525(3) . ?
C5 H5A 0.9700 . ?
C5 H5B 0.9700 . ?
C6 H6A 0.9700 . ?
C6 H6B 0.9700 . ?
```

```
C8 C9 1.320(4) . ?
C8 C10 1.493(4) . ?
C9 H9A 0.9300 . ?
C9 H9B 0.9300 . ?
C10 H10A 0.9600 . ?
C10 H10B 0.9600 . ?
C10 H10C 0.9600 . ?

loop_
 _geom_angle_atom_site_label_1
 _geom_angle_atom_site_label_2
 _geom_angle_atom_site_label_3
 _geom_angle
 _geom_angle_site_symmetry_1
 _geom_angle_site_symmetry_3
 _geom_angle_publ_flag
C7 C1 C6 114.16(15) . . ?
C7 C1 C2 108.13(15) . . ?
C6 C1 C2 110.55(15) . . ?
C7 C1 H1A 107.9 . . ?
C6 C1 H1A 107.9 . . ?
C2 C1 H1A 107.9 . . ?
C7 O2 H2 112(2) . . ?
C3 C2 C1 111.49(15) . . ?
C3 C2 H2A 109.3 . . ?
C1 C2 H2A 109.3 . . ?
C3 C2 H2B 109.3 . . ?
C1 C2 H2B 109.3 . . ?
H2A C2 H2B 108.0 . . ?
C3 O3 H3 111.0(19) . . ?
O3 C3 C2 107.39(14) . . ?
O3 C3 C4 110.78(14) . . ?
C2 C3 C4 110.76(14) . . ?
O3 C3 H3A 109.3 . . ?
C2 C3 H3A 109.3 . . ?
C4 C3 H3A 109.3 . . ?
C8 C4 C3 113.59(14) . . ?
C8 C4 C5 112.60(16) . . ?
C3 C4 C5 109.10(16) . . ?
C8 C4 H4A 107.1 . . ?
C3 C4 H4A 107.1 . . ?
C5 C4 H4A 107.1 . . ?
C6 C5 C4 111.50(16) . . ?
C6 C5 H5A 109.3 . . ?
```

```
C4 C5 H5A 109.3 . . ?
C6 C5 H5B 109.3 . . ?
C4 C5 H5B 109.3 . . ?
H5A C5 H5B 108.0 . . ?
C1 C6 C5 111.03(17) . . ?
C1 C6 H6A 109.4 . . ?
C5 C6 H6A 109.4 . . ?
C1 C6 H6B 109.4 . . ?
C5 C6 H6B 109.4 . . ?
H6A C6 H6B 108.0 . . ?
O1 C7 O2 122.17(18) . . ?
O1 C7 C1 123.82(16) . . ?
O2 C7 C1 113.93(16) . . ?
C9 C8 C10 121.8(2) . . ?
C9 C8 C4 120.7(2) . . ?
C10 C8 C4 117.4(2) . . ?
C8 C9 H9A 120.0 . . ?
C8 C9 H9B 120.0 . . ?
H9A C9 H9B 120.0 . . ?
C8 C10 H10A 109.5 . . ?
C8 C10 H10B 109.5 . . ?
H10A C10 H10B 109.5 . . ?
C8 C10 H10C 109.5 . . ?
H10A C10 H10C 109.5 . . ?
H10B C10 H10C 109.5 . . ?

loop_
 _geom_torsion_atom_site_label_1
 _geom_torsion_atom_site_label_2
 _geom_torsion_atom_site_label_3
 _geom_torsion_atom_site_label_4
 _geom_torsion
 _geom_torsion_site_symmetry_1
 _geom_torsion_site_symmetry_2
 _geom_torsion_site_symmetry_3
 _geom_torsion_site_symmetry_4
 _geom_torsion_publ_flag
C7 C1 C2 C3 -178.81(14) . . . . ?
C6 C1 C2 C3 55.55(19) . . . . ?
C1 C2 C3 O3 -178.32(13) . . . . ?
C1 C2 C3 C4 -57.24(19) . . . . ?
O3 C3 C4 C8 -56.9(2) . . . . ?
C2 C3 C4 C8 -175.98(17) . . . . ?
O3 C3 C4 C5 176.58(15) . . . . ?
```

```
C2 C3 C4 C5 57.5(2) . . . . ?
C8 C4 C5 C6 175.17(18) . . . . ?
C3 C4 C5 C6 -57.8(2) . . . . ?
C7 C1 C6 C5 -177.26(17) . . . . ?
C2 C1 C6 C5 -55.1(2) . . . . ?
C4 C5 C6 C1 57.3(2) . . . . ?
C6 C1 C7 O1 -153.2(2) . . . . ?
C2 C1 C7 O1 83.3(2) . . . . ?
C6 C1 C7 O2 29.9(2) . . . . ?
C2 C1 C7 O2 -93.6(2) . . . . ?
C3 C4 C8 C9 117.6(2) . . . . ?
C5 C4 C8 C9 -117.8(3) . . . . ?
C3 C4 C8 C10 -62.9(3) . . . . ?
C5 C4 C8 C10 61.7(3) . . . . ?

_refine_diff_density_max    0.180
_refine_diff_density_min   -0.123
_refine_diff_density_rms    0.028
```
