# Supplementary material for: Biotransformation of (–)-Isopulegol by Rhodococcus rhodochrous
Source: Pharmaceuticals (Basel). 2022 Aug 3;15(8):964. doi: 10.3390/ph15080964 (PMC9412403; doi:10.3390/ph15080964)
Supplement: Supplementary file 1 [file pharmaceuticals-15-00964-s001.zip › pharmaceuticals-1821943-supplementary/pharmaceuticals-1821943-supplementary.pdf]

Supplementary Material (Ivshina et al., 2022)

Procedure of cell staining by Nile red fluorescent dye (Figure 2).

To detect intracellular lipid inclusions, bacterial cells were stained with a 0.08% Nile Red solution in dimethyl sulfoxide (Nanjing Dulai Biotechnology Co., Nanjing, China) as previously described (Mrunalini and Girisha, 2017). For this, the cell suspension (1 mL) was centrifuged at 12,000 rpm for 5 min. The precipitated cells were resuspended in 1 mL of distilled water and supplemented with 40 μL of the working solution of Nile Red (0.3 μg/mL, final concentration). The resulting suspension was incubated at 28 °C for 40 min with shaking at 160 rpm. Cells were separated from the reaction medium by centrifugation and resuspended in 1 mL of distilled water. The cell suspension (5 μL) was spread on a clean glass slide, and fluorescence was read at two spectral settings on an Axio Imager M2 microscope (Carl Zeiss Microscopy GmbH, Jena, Germany): yellow-gold fluorescence using a 450–500 nm band pass exciter filter and red fluorescence using a 515–560 nm band pass exciter filter.

Procedure of cell staining by Live/Dead fluorescent dye (Figure 4).

To differentiate living and dead cells, the bacterial suspensions were stained with the Live/Dead® BacLight™ Bacterial Viability Kit fluorescent dye (Invitrogen, Carlsbad, CA, USA), air dried in the dark for 10–15 min, and washed with deionized water to remove residual dye. The cells were visualized on CLSM Olympus FV1000 (Olympus Corporation, Tokyo, Japan). To excite the fluorescence of SYTO 9 and propidium iodide, an argon laser (λ = 488 nm) with a 505/525 nm barrier filter and a helium neon laser (λ = 543 nm) with a 560/660 nm barrier filter were used, respectively.

Table S1. Morphometric parameters of cells of *R. rhodochrous* IEGM 1362

**Conditions**	**Length, μm**	**Width, μm**	**Area, μm^2**	**Volume, μm^3**	**Relative area, μm^{-1}**
Biotic control	1.58 ± 0.22	1.12 ± 0.14	4.74 ± 0.69	1.55 ± 0.31	3.06 ± 0.26
(–)-Isopulegol	1.33 ± 0.26	1.15 ± 0.09	4.50 ± 0.33	1.39 ± 0.16	3.24 ± 0.21

HNTs	1.56 ± 0,40	1.04 ± 0,11	4.20 ± 0.59	1.29 ± 0.29	3.26 ± 0.29
(–)-Isopulegol and HNTs	1.82 ± 0.48	1.06 ± 0.14	4.76 ± 0.92	1.59 ± 0.49	2.99 ± 0.31

Table S2. Parameters of cell surface of *R. rhodochrous* IEGM 1362

Conditions	Electrokinetic potential, mV	Roughness, nm
Biotic control	-36.8 ± 6.90	151.4 ± 10.3
(–)-Isopulegol	-33.3 ± 8.00	231.5 ± 83.2
HNTs	-32.8 ± 6.95	178.3 ± 8.9
(–)-Isopulegol and HNTs	-36.7 ± 7.82	218.2 ± 25.4

Table S3. *Rhodococcus* strains used in the research

Species	No. of strains	Strain number in the IEGM collection
R. erythropolis	3	IEGM 199, IEGM 201, IEGM 344
R. fascians	2	IEGM 525, IEGM 1218
R. jostii	1	IEGM 60
R. opacus	1	IEGM 488
R. qingshengii	3	IEGM 247, IEGM 267, IEGM 1016^{T}
R. rhodochrous	20	IEGM 63, IEGM 64, IEGM 66, IEGM 67, IEGM 107, IEGM 608, IEGM 609, IEGM 629, IEGM 632, IEGM 639, IEGM 646, IEGM 647, IEGM 653, IEGM 655, IEGM 654, IEGM 757, IEGM 760, IEGM 1138, IEGM 1162, IEGM 1362
R. ruber	10	IEGM 70^{T}, IEGM 71, IEGM 72, IEGM 73, IEGM 74, IEGM 76, IEGM 77, IEGM 79, IEGM 80, IEGM 81
